# Straight Versus Branched Chain Substituents in 4′-(Butoxyphenyl)-3,2′:6′,3″-terpyridines: Effects on (4,4) Coordination Network Assemblies

**DOI:** 10.3390/polym12081823

**Published:** 2020-08-14

**Authors:** Dalila Rocco, Alessandro Prescimone, Edwin C. Constable, Catherine E. Housecroft

**Affiliations:** Department of Chemistry, University of Basel, BPR 1096, Mattenstrasse 24a, CH-4058 Basel, Switzerland; dalila.rocco@unibas.ch (D.R.); alessandro.prescimone@unibas.ch (A.P.); edwin.constable@unibas.ch (E.C.C.)

**Keywords:** coordination network, 3,2′:6′,3″-terpyridine, cobalt(II) thiocyanate, isomers, crystal structure

## Abstract

The preparation and characterization of the isomers *rac*-4′-(4-butan-2-yloxyphenyl)-3,2′:6′,3″-terpyridine (*rac*-**2**), 4′-(2-methylpropoxyphenyl)-3,2′:6′,3″-terpyridine (**3**) and 4′-(*tert*-butoxyphenyl)-3,2′:6′,3″-terpyridine (**4**) are reported. The compounds react with Co(NCS)_2_ under conditions of crystal growth at room temperature to give single crystals of [{Co(*rac*-**2**)_2_(NCS)_2_}·CHCl_3_]*_n_*, [Co(**3**)_2_(NCS)_2_]*_n_* and [{Co(**4**)_2_(NCS)_2_}·CHCl_3_]*_n_* which possess (4,4) networks, with the Co centers acting as 4-connecting nodes. Powder X-ray diffraction (PXRD) was used to confirm that the crystals chosen for single crystal X-ray diffraction were representative of the bulk samples. The detailed structures of the three networks have been compared with that of the previously reported [{Co(**1**)_2_(NCS)_2_}·4CHCl_3_]*n* in which **1** is 4′-(butoxyphenyl)-3,2′:6′,3″-terpyridine. Whereas the switch from **1** with the straight-chain butoxy substituent to *rac*-**2**, **3** and **4** with branched chains causes significant structural perturbation, changes in the spatial properties of the branched substituents are accommodated with subtle conformational changes in the 3,2′:6′,3″-tpy domain.

## 1. Introduction

**Scheme 1:**
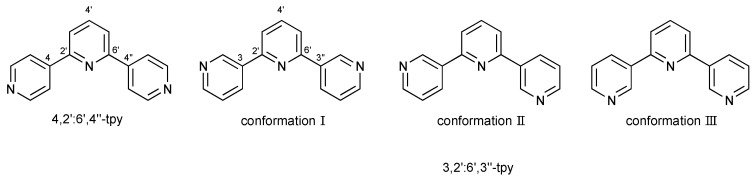
The structures of 4,2′:6′,4″- and 3,2′:6′,3″-terpyridine showing different planar conformations of 3,2′:6′,3″-tpy.

Over the last few years, interest in the use of ditopic 4,2′:6′,4″- and 3,2′:6′,3″-terpyridine (4,2′:6′,4″-tpy and 3,2′:6′,3″-tpy) ligands as building blocks in the assembly of coordination polymers and networks has increased [1,2,3]. In the absence of additional coordination sites such as carboxylate, the role of a 4,2′:6′,4″- or 3,2′:6′,3″-tpy ligand in a coordination assembly is typically restricted to that of a ditopic linker between two metal centers. To date, there are no examples in which the central pyridine ring coordinates to a metal center. On the one hand, the outcome of the assembly process with 4,2′:6′,4″- and 3,2′:6′,3″-tpy ligands is less predictable than with a linear rigid-rod such as 4,4′-bipyridine [4,5]. This is particularly true for 3,2′:6′,3″-tpy, where the conformational flexibility leads to varying vectorial dispositions of the nitrogen lone pairs (Scheme 1) [6,7]. On the other hand, the synthetic ease with which substituents can be introduced into the 4′-position (Scheme 1) using either the Kröhnke approach [8] or the one-pot strategy of Wang and Hanan [9] means that a wide ranging suite of ligands with 4,2′:6′,4″- and 3,2′:6′,3″-tpy metal-binding domains can be easily accessed. Among these are tetratopic bis(4,2′:6′,4″)- and bis(3,2′:6′,3″)-terpyridines, which act as 4-connecting nodes and can be used to increase the dimensionality of the assembly [3,10].

We commented above that 4,2′:6′,4″- and 3,2′:6′,3″-tpy ligands lacking additional coordination sites usually act as ditopic linkers in coordination assemblies. For example, (4,4) nets are the typical outcome of reactions between Co(NCS)_2_ (which acts as a 4-connecting node) and 4,2′:6′,4″- and 3,2′:6′,3″-tpy ligands [6,11,12,13,14,15]. However, remarkably, 4,2′:6′,4″-tpy ligands bearing 4′-hexyloxy and 4′-nonyloxy substituents combine with Co(NCS)_2_ to assemble uninodal, 3-dimensional, chiral nets [16]. In this exceptional case, the long alkyloxy chains appear to stabilize the 3-dimensional network, by threading through the cavities in the assembly. This is somewhat reminiscent of the role played by long alkyloxy chains in stabilizing 2D→2D parallel interpenetrated sheets in [Zn_2_Cl_4_(L)]*_n_* networks, where L = 1,4-bis(hexyloxy)-2,5-bis(4,2′:6′,4″-terpyridin-4′-yl)benzene, 1,4-bis(octyloxy)-2,5-bis(4,2′:6′,4″-terpyridin-4′-yl)benzene or 1,4-bis(decyloxy)-2,5-bis(4,2′:6′,4″-terpyridin-4′-yl)benzene [17,18,19]. To understand the assembly algorithms, systematic investigations are essential, and yet, they remain relatively rare. We have contributed to such understanding by investigating the effects of systematically increasing the lengths of straight-chain 4′-alkyoxy groups in 4′-alkyoxyphenyl-4,2′:6′,4″-terpyridine and 4′-alkyoxyphenyl-3,2′:6′,3″-terpyridine ligands, on the assembly of zinc(II) and cobalt(II) coordination networks [6,20].

**Scheme 2:**
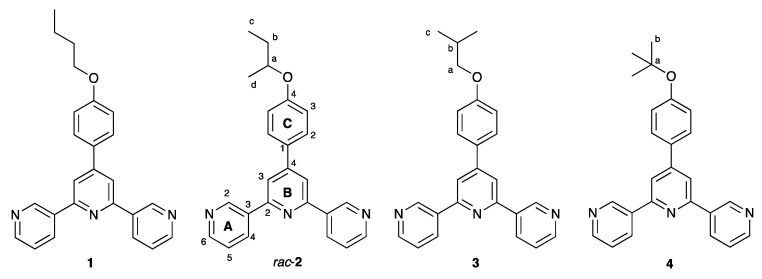
Structures of the isomeric ligands **1**–**4** with numbering scheme used for NMR spectroscopic assignments. In *rac*-**2**, C^a^ is a stereogenic center.

**Figure 1:**
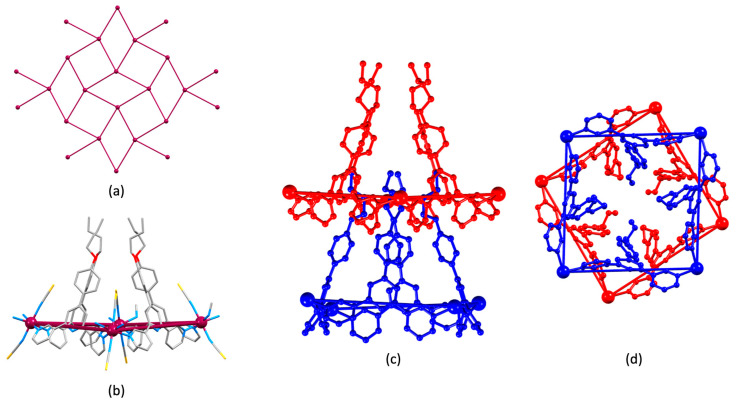
(**a**) Part of one (4,4) net in [{Co(**1**)_2_(NCS)_2_}·4CHCl_3_]*n*; (**b**) the cone-like arrangement of four ligands **1** around the edges of one of two geometrically different rhombuses; (**c**) and (**d**) the relationship between the rhombuses shown in diagram (**b**) in adjacent sheets [6].

In the present work, we consider the isomeric set of ligands **1**–**4** shown in Scheme 2. We have previously reported that the reaction of Co(NCS)_2_ with **1** under conditions of crystal growth by layering yields a (4,4) net comprising two geometrically different rhombuses (Figure 1a). In one type of rhombus, all four ligand-linkers are directed towards the same side of the rhombus, with the butoxy chains in extended conformations forming a cone-like array (Figure 1b). Each cone in one {4,4} net is accommodated within a similar cone in the next sheet (Figure 1c,d) [6]. We now describe how the network responds to a change from butoxy to the isomeric butan-2-yloxy, 2-methylpropoxy and *tert*-butoxy substituents.

## 2. Materials and Methods

### 2.1. General

^1^H, ^13^C{^1^H} and 2D NMR spectra were recorded on a Bruker Avance iii-500 spectrometer (Bruker BioSpin AG, Fällanden, Switzerland) at 298 K. The ^1^H and ^13^C NMR chemical shifts were referenced with respect to residual solvent peaks (*δ* TMS = 0). A Shimadzu LCMS-2020 instrument (Shimadzu Schweiz GmbH, 4153 Reinach, Switzerland) was used to record electrospray ionization (ESI) mass spectra. A PerkinElmer UATR Two instrument (PerkinElmer, 8603 Schwerzenbach, Switzerland) was used to record FT-infrared (IR) spectra, and Shimadzu UV2600 (Shimadzu Schweiz GmbH, 4153 Reinach, Switzerland) or Cary 5000 (Agilent Technologies AG, 4052 Basel, Switzerland) spectrophotometers were used to record absorption spectra. 3-Acetylpyridine and 4-hydroxybenzaldehyde were purchased from Acros Organics (Fisher Scientific AG, 4153 Reinach, Switzerland), *rac*-4-(butan-2-yloxy)benzaldehyde, 4-(2-methylpropoxy)benzaldehyde and 4-(*tert*-butoxy)benzaldehyde from Fluorochem (Chemie Brunschwig AG, 4052 Basel, Switzerland), 1-bromo-2-methylpropane from Sigma Aldrich (Chemie Brunschwig AG, 4052 Basel, Switzerland), and were used as received. 4′-(4-Hydroxyphenyl)-3,2′:6′,3″-terpyridine was prepared as previously described [6].

### 2.2. Compound rac-***2***

*rac*-4-(Butan-2-yloxy)benzaldehyde (1.00 g, 5.61 mmol) was dissolved in EtOH (40 mL), and then 3-acetylpyridine (1.36 g, 1.24 mL, 11.2 mmol) and crushed KOH (0.630 g, 11.2 mmol) were added to the solution. Aqueous NH_3_ (32%, 20.0 mL) was slowly added to the reaction mixture. This was stirred at room temperature (ca. 22 °C) overnight, during which time a small amount of solid formed. The solvent was removed under vacuum, and the solid residue was washed with water (3 × 10 mL), recrystallized from MeOH/H_2_O and dried in vacuo. Compound *rac*-**2** was isolated as a colorless powder (0.531 g, 1.39 mmol, 24.8%). M.p. = 161 °C. ^1^H NMR (500 MHz, CDCl_3_): *δ*/ppm 9.37 (d, *J* = 1.7 Hz, 2H, H^A2^), 8.70 (dd, *J* = 4.7, 1.4 Hz, 2H, H^A6^), 8.51 (dt, *J* = 9.8, 1.8 Hz, 2H, H^A4^), 7.92 (s, 2H, H^B3^), 7.69 (m, 2H, H^C2^), 7.46 (m, 2H, H^A5^), 7.05 (m, 2H, H^C3^), 4.41 (m, 1H, H^a^), 1.81 (m, 1H, H^b1^), 1.68 (m, 1H, H^b2^), 1.36 (m, 3H, H^d^), 1.02 (t, *J* = 7.5 Hz, 3H, H^c^). ^13^C{^1^H} NMR (500 MHz, CDCl_3_): *δ*/ppm 159.8 (C^C4^), 155.4 (C^A3^), 150.6 (C^B4^), 150.2 (C^A6^), 148.5 (C^A2^), 135.0 (C^A4^), 134.7 (C^B2^), 130.1 (C^C1^), 128.5 (C^C2^), 123.8 (C^A5^), 117.3 (C^B3^), 116.6 (C^C3^), 75.4 (C^a^), 29.3 (C^b^), 19.4 (C^d^), 9.9 (C^c^). UV-VIS (MeCN, 2.0 × 10^–5^ mol dm^–3^) λ/nm 227 (ε/dm^3^ mol^–1^ cm^–1^ 35,100), 273 (33,100). ESI-MS *m/z* 382.19 [M + H]^+^ (calc. 382.19). Found C 78.65, H 6.13, N 10.85; required for C_25_H_23_N_3_O C 78.71, H 6.08, N 11.02.

### 2.3. Compound ***4***

4-(*tert*-Butoxy)benzaldehyde (1.78 g, 1.75 mL, 10.0 mmol) was dissolved in EtOH (50 mL), then 3-acetylpyridine (2.42 g, 2.20 mL, 20.0 mmol) and crushed KOH (1.12 g, 20.0 mmol) were added to the solution. Aqueous NH_3_ (32%, 38.5 mL) was slowly added to the reaction mixture, and this was stirred at room temperature overnight. The solid that formed was collected by filtration, washed with H_2_O (3 × 10 mL) and EtOH (3 × 10 mL), recrystallized from EtOH and dried in vacuo. Compound **4** was isolated as a colorless powder (0.781 g, 2.05 mmol, 20.5%). M.p. = 207 °C. ^1^H NMR (500 MHz, CDCl_3_): *δ*/ppm 9.37 (d, 2H, H^A2^), 8.70 (dd, *J* = 4.8, 1.6 Hz, 2H, H^A6^), 8.51 (m, 2H, H^A4^), 7.93 (s, 2H, H^B3^), 7.67 (m, 2H, H^C2^), 7.45 (m, 2H, H^A5^), 7.16 (m, 2H, H^C3^), 1.43 (s, 9H, H^b^). ^13^C{^1^H} NMR (500 MHz, CDCl_3_): *δ*/ppm 157.2 (C^C4^), 155.4 (C^A3^), 150.6 (C^B4^), 150.3 (C^A6^), 148.5 (C^A2^), 134.9 (C^A4^), 134.7 (C^B2^), 132.9 (C^C1^), 127.9 (C^C2^), 124.6 (C^A5^), 123.7 (C^B3^), 117.5 (C^C3^), 79.4 (C^a^), 29.1 (C^b^). UV-VIS (MeCN, 2.0 × 10^–5^ mol dm^−3^) λ/nm 224 (ε/dm^3^ mol^−1^ cm^−1^ 32,550), 264 (38,600), 315 sh (10,800). ESI^–^MS *m/z* 382.17 [M + H]^+^ (calc. 382.19). Found C 78.60, H 5.98, N 10.94; required for C_25_H_23_N_3_O C 78.71, H 6.08, N 11.02.

### 2.4. Compound ***3a***

4-(2-Methylpropoxy)benzaldehyde (1.78 g, 1.75 mL, 10.0 mmol) was dissolved in EtOH (40 mL), and then 3-acetylpyridine (2.42 g, 2.20 mL, 20.0 mmol) and crushed KOH (1.12 g, 20.0 mmol) were added to the solution. Aqueous NH_3_ (32%, 38.5 mL) was slowly added to the reaction mixture, which was then stirred at room temperature overnight. The solid that formed was collected by filtration, washed with water (3 × 10 mL), EtOH (3 × 10 mL), recrystallized from EtOH and dried in vacuo. Compound **3a** was isolated as a colorless powder (0.167 g, 0.244 mmol, 4.9%). M.p. = 203 °C. ^1^H NMR (500 MHz, CDCl_3_): *δ*/ppm 9.04 (d, *J* = 2.3 Hz, 1H, H^C2^), 8.76 (d, *J* = 2.3 Hz, 1H, H^B2^), 8.52 (dd, *J* = 4.8, 1.7 Hz, 1H, H^B6^), 8.42 (dd, *J* = 4.8, 1.7 Hz, 1H, H^A6^), 8.38–8.34 (overlapping m, 2H, H^C6 + A2^), 8.04 (dt, *J* = 8.0, 2.0 Hz, 1H, H^C4^), 7.71 (dt, *J* = 8.1, 2.0 Hz, 1H, H^B4^), 7.42 (dt, *J* = 8.1, 2.0 Hz, 1H, H^A4^), 7.20 (m, 1H, H^C5^), 7.10 (m, 1H, H^B5^), 7.06 (m, 2H, H^D2^), 7.00 (m, 1H, H^A5^), 6.96 (m, 2H, H^E2^), 6.61 (m, 2H, H^D3^), 6.39 (m, 2H, H^E3^), 5.62 (d, *J* = 11.9 Hz, 1H, H^2^), 5.03 (d, *J* = 2.4 Hz, 1H, H^OH^), 4.29 (dd, *J* = 4.7, 4.7 Hz, 1H, H^4^), 4.12–4.03 (m, 2H, H^3 + 5^), 3.53 (m, 2H, H^a’^), 3.39 (m, 2H, H^a^), 3.21 (m, 1H, H^6ax^), 2.03 (dd, *J* = 13.8, 3.5 Hz, 1H, H^6eq^), 1.93 (m, 1H, H^b’^), 1.85 (m, 1H, H^b^), 0.93 (d, *J* = 6.7 Hz, 6H, H^c’^), 0.86 (d, *J* = 6.7 Hz, 6H, H^c^). ^13^C{^1^H} NMR (500 MHz, CDCl_3_): *δ*/ppm 206.9 (C^CO2^), 206.1 (C^CO1^), 158.5 (C^E4^), 158.3 (C^D4^), 153.6 (C^B6^), 152.5 (C^A6^), 149.7 (C^B2^), 149.2 (C^A2^), 148.7 (C^C6^), 147.1 (C^C2^), 142.1 (C^C3^), 135.2 (C^B4^), 135.0 (C^A3^), 134.8 (C^A4^), 133.1 (overlapping C^C4 + B3^), 132.7 (C^D1^), 130.3 (C^E1^), 129.7 (C^E2^), 128.6 (C^D2^), 123.3 (C^C5^), 123.2 (C^B5^), 122.9 (C^A5^), 114.7 (C^D3^), 114.6 (C^E3^), 75.0 (C^1^), 74.5 (C^a’^), 74.3 (C^a^), 53.6 (C^4^), 51.1 (C^2^), 46.8 (C^3^), 41.2 (C^5^), 38.8 (C^6^), 28.3 (C^b’^), 28.2 (C^b^), 19.3 (C^c’^), 19.2 (C^c^). UV-VIS (MeCN, 2.0 × 10^–5^ mol dm^–3^) λ/nm 228 (ε/dm^3^ mol^–1^ cm^–1^ 53,320), 263 (13,950). ESI-MS *m/z* 684.28 [M + H]^+^ (calc. 684.34). Found C 75.08, H 6.54, N 5.97; required for C_43_H_45_N_3_O_5_ C 75.52, H 6.63, N 6.14.

### 2.5. Compound ***3***

4′-(4-Hydroxyphenyl)-3,2′:6′,3″-terpyridine (1.26 g, 3.86 mmol) was dissolved in DMF (40 mL) and the solution was heated to 80 °C. Then, anhydrous K_2_CO_3_ (1.60 g, 11.6 mmol) was added, and the color changed from yellow to red-brown. After 5 min, 1-bromo-2-methylpropane (0.582 g, 0.46 mL, 4.25 mmol) was added to the reaction mixture. This was stirred at 80 °C overnight, then it was cooled to room temperature and poured into ice water and stirred for 20 min, but no solid was formed. Extraction with CHCl_3_ and washings with aqueous K_2_CO_3_ solution were performed. The organic layers were dried over MgSO_4_, and the solvent was then removed. The solid product was purified by column chromatography (SiO_2_, ethyl acetate: cyclohexane 3:1, R_f_ = 0.25). Compound **3** was obtained as a pale yellow solid (0.282 g, 0.739 mmol, 19.2%). M.p. = 115 °C. ^1^H NMR (500 MHz, CDCl_3_): *δ*/ppm 9.33 (d, *J* = 1.5 Hz, 2H, H^A2^), 8.65 (dd, *J* = 4.8, 1.7 Hz, 2H, H^A6^), 8.44 (dt, *J* = 8.0, 2.0 Hz, 2H, H^A4^), 7.84 (s, 2H, H^B3^), 7.64 (m, 2H, H^C2^), 7.40 (m, 2H, H^A5^), 7.01 (m, 2H, H^C3^), 3.76 (d, *J* = 6.5 Hz, 2H, H^a^), 2.11 (m, 1H, H^b^), 1.04 (d, *J* = 6.7 Hz, 6H, H^c^). ^13^C{^1^H} NMR (500 MHz, CDCl_3_): *δ*/ppm 160.6 (C^C4^), 155.2 (C^A3^), 150.3 (C^B4^), 150.1 (C^A6^), 148.4 (C^A2^), 134.8 (C^B2^), 134.5 (C^A4^), 130.0 (C^C1^), 128.3 (C^C2^), 123.6 (C^A5^), 117.1 (C^B3^), 115.3 (C^C3^), 74.6 (C^a^), 28.3 (C^b^), 19.3 (C^c^). UV-VIS (MeCN, 2.0 × 10^−5^ mol dm^−3^) λ/nm 227 (ε/dm^3^ mol^−1^ cm^−1^ 39,700), 269 (37,450). ESI-MS *m/z* 382.14 [M + H]^+^ (calc. 382.19). Found C 78.40, H 5.99, N 10.88; required for C_25_H_23_N_3_O C 78.71, H 6.08, N 11.02.

### 2.6. Crystal Growth of [{Co(rac-***2***)_2_(NCS)_2_}·CHCl_3_]_n_

A solution of Co(NCS)_2_ (5.3 mg, 0.030 mmol) in MeOH (5 mL) was layered over a CHCl_3_ solution (4 mL) of compound *rac*-**2** (11.4 mg, 0.030 mmol), in a crystallization tube (i.d. = 13.6 mm, 24 mL). Colorless block-like crystals grew after 6 days, and a single crystal was selected for X-ray diffraction. Single-crystal structure determination confirmed a formulation of [{Co(*rac*-**2**)_2_(NCS)_2_}·CHCl_3_]*_n_*. The remaining crystals in the tube were washed with MeOH and CHCl_3_, dried under vacuum and analyzed by PXRD and FT-IR spectroscopy.

### 2.7. Crystal Growth of [Co(***3***)_2_(NCS)_2_]_n_

A solution of Co(NCS)_2_ (5.3 mg, 0.030 mmol) in MeOH (6 mL) was layered over a CHCl_3_ solution (6 mL) of compound **3** (11.4 mg, 0.030 mmol), in a crystallization tube (i.d. = 13.6 mm, 24 mL). Pink block-like crystals grew within a period of 15 days. A crystal suitable for single-crystal X-ray diffraction was selected, and the remaining crystals in the tube were washed with MeOH and CHCl_3_, dried under vacuum and analyzed by powder X-ray diffraction (PXRD) and FT-IR spectroscopy. The single-crystal structure confirmed a formulation of [Co(**3**)_2_(NCS)_2_]*_n_*.

### 2.8. Crystal Growth of [{Co(***4***)_2_(NCS)_2_}·CHCl_3_]_n_

A solution of Co(NCS)_2_ (5.3 mg, 0.030 mmol) in MeOH (8 mL) was layered over a CHCl_3_ solution (8 mL) of **4** (11.4 mg, 0.030 mmol) in a crystallization tube (i.d. = 13.6 mm, 24 mL). Pink plate-like crystals grew over a period of 40 days, and a single crystal was selected for single-crystal X-ray diffraction and structure determination showed the formation of [{Co(**4**)_2_(NCS)_2_}·CHCl_3_]*_n_*. The remaining crystals in the tube were washed with MeOH and CHCl_3_, and after drying under vacuum, they were analyzed by FT-IR spectroscopy and PXRD.

### 2.9. Crystallography

Single crystal data were collected on a Bruker APEX-II diffractometer (CuKα radiation) with data reduction, solution and refinement, using the programs APEX [21], ShelXT [22], Olex2 [23] and ShelXL v. 2014/7 [24], or using a STOE StadiVari diffractometer equipped with a Pilatus300K detector and with a Metaljet D2 source (GaKα radiation), and solving the structure using Superflip [25,26] and Olex2 [23]; the model was refined with ShelXL v. 2014/7 [24]. Structure analysis, including the ORTEP representations, used CSD Mercury 2020.1 [27]. In [{Co(*rac*-**2**)_2_(NCS)_2_}·CHCl_3_]*_n_* and [{Co(**4**)_2_(NCS)_2_}·CHCl_3_]*_n_*, SQUEEZE [28] was used to treat the solvent region and, in each case, the electron density removed equated to one CHCl_3_ molecule per CoL_2_(NCS)_2_ unit; formulae and numbers were appropriately adjusted.

Powder X-ray diffraction (PXRD) patterns were collected at room temperature in transmission mode, using a Stoe Stadi P diffractometer equipped with a Cu Kα1 radiation (Ge(111) monochromator) and a DECTRIS MYTHEN 1K detector. The reflections of the bulk samples of [{Co(*rac*-**2**)_2_(NCS)_2_}·CHCl_3_]*_n_*, [Co(**3**)_2_(NCS)_2_]*_n_* and [{Co(**4**)_2_(NCS)_2_}·CHCl_3_]*_n_* were each indexed with the monoclinic cells *P*2_1_/*n*. A profile matching analysis [29,30,31] of the diffraction patterns was performed with the package FULLPROF SUITE [31,32] (version May-2020) using a previously determined instrument resolution function based on a NIST640d standard. The structural models were taken from the single crystal X-ray diffraction refinements. Refined parameters in profile matching were: zero shift, lattice parameters, scale factor, coordinates of the S atoms, preferred orientations, peak asymmetry, sample transparency, and peaks shapes as a Thompson-Cox-Hastings pseudo-Voigt function. The refinements confirmed that the bulk samples were consistent with the single crystal structures for all the compounds.

### 2.10. [{Co(rac-***2***)_2_(NCS)_2_}·CHCl_3_]_n_

C_53_H_47_Cl_3_CoN_8_O_2_S_2_, *M*_r_ = 1057.38, colorless block, monoclinic, space group *P*2_1_/*n*, *a* = 13.2404(9), *b* = 13.2088(9), *c* = 15.0375(10) Å, β = 99.261(3)°, *V* = 2595.6(3) Å^3^, *D*_c_ = 1.353 g cm^−3^, *T* = 150 K, *Z* = 2, μ(Cu*Kα*) = 5.151 mm^−1^. Total 17,402 reflections, 4804 unique (*R*_int_ = 0.0309). Refinement of 4518 reflections (277 parameters) with *I* > 2*σ*(*I*) converged at final *R*_1_ = 0.0772 (*R*_1_ all data = 0.0801), *wR*_2_ = 0.2350 (*wR*2 all data = 0.2394), gof = 1.097. CCDC 2013944.

### 2.11. [Co(***3***)_2_(NCS)_2_]_n_

C_52_H_46_CoN_8_O_2_S_2_, *M*_r_ = 938.02, pink block, monoclinic, space group *P*2_1_/*n*, *a* = 9.6250(5), *b* = 16.3159(6), *c* = 14.7532(7) Å, β = 104.178(4)°, *V* = 2246.28(18) Å^3^, *D*_c_ = 1.387 g cm^−3^, *T* = 150 K, *Z* = 2, μ(Ga*Kα*) = 2.896 mm^−1^. Total 30,956 reflections, 4723 unique (*R*_int_ = 0.0465). Refinement of 4086 reflections (297 parameters) with *I* > 2*σ*(*I*) converged at final *R*_1_ = 0.0702 (*R*_1_ all data = 0.0869), *wR*_2_ = 0.1608 (*wR*2 all data = 0.1763), gof = 1.168. CCDC 2013945.

### 2.12. [{Co(***4***)_2_(NCS)_2_}·CHCl_3_]_n_

C_53_H_47_Cl_3_CoN_8_O_2_S_2_, *M*_r_ = 1057.44, pink plate, monoclinic, space group *P*2_1_/*n*, *a* = 13.0484(7), *b* = 13.1230(5), *c* = 15.3008(8) Å, β = 99.454(4)°, *V* = 2584.4(2) Å^3^, *D*_c_ = 1.359 g cm^−3^, *T* = 150 K, *Z* = 2, μ(Ga*Kα*) = 3.470 mm^−1^. Total 33,416 reflections, 5439 unique (*R*_int_ = 0.0756). Refinement of 5002 reflections (298 parameters) with *I* > 2*σ*(*I*) converged at final *R*_1_ = 0.0733 (*R*_1_ all data = 0.0798), *wR*_2_ = 0.1593 (*wR*2 all data = 0.1626), gof = 1.0505. CCDC 2013946.

## 3. Results and Discussion

### 3.1. Synthesis and Characterization of Compounds rac-***2***, ***3*** and ***4***

**Scheme 3:**
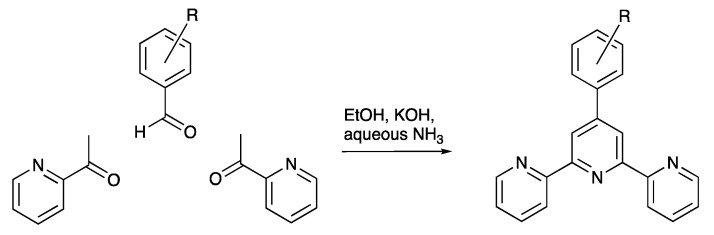
The Wang and Hanan one-pot approach to 4′-aryl-2,2′:6′,2″-terpyridines [9].

**Scheme 4:**
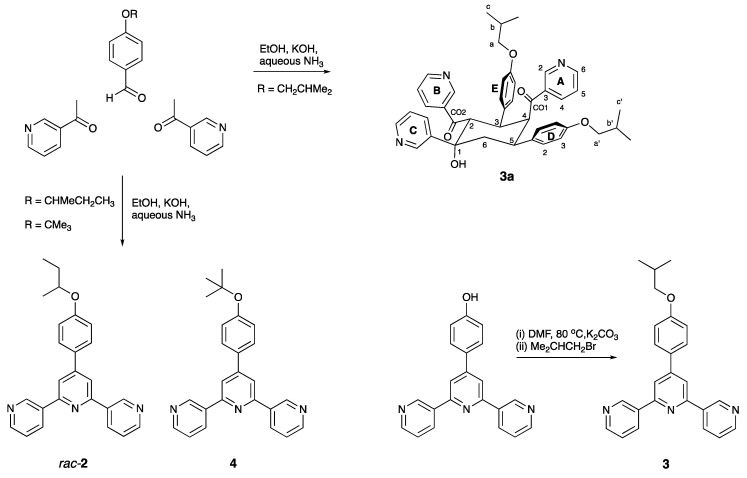
Synthetic routes to compounds *rac*-**2**, **3** and **4**, and the formation of the cyclic product **3a** (with numbering scheme for the NMR characterization).

**Figure 2:**
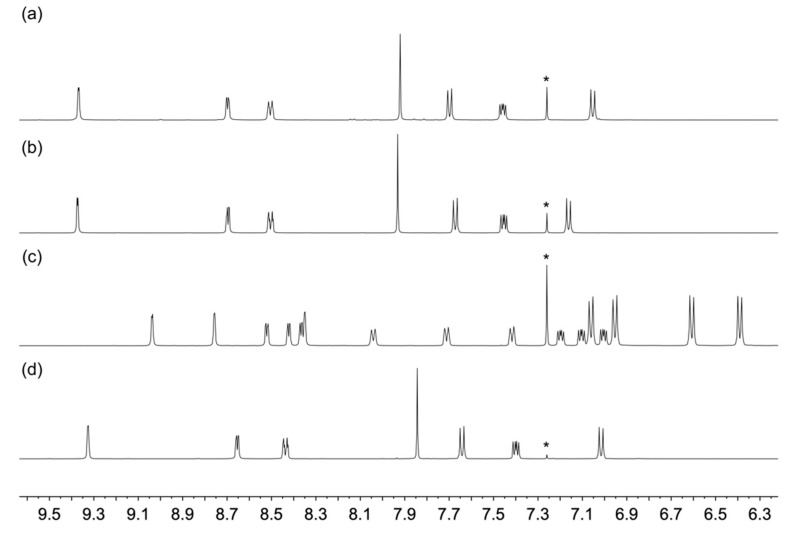
Comparison of the aromatic regions of the ^1^H NMR spectra (500 MHz, 298 K, CDCl_3_) of (**a**) *rac*-**2**, (**b**) **4**, (**c**) **3a** and (**d**) **3**. * = residual CHCl_3_.

The one-pot method developed by Wang and Hanan [9] for the preparation of 4′-aryl-2,2′:6′,2″-terpyridines (Scheme 3) is readily adapted to the synthesis of 4′-aryl-3,2′:6′,3″-terpyridines, by replacing the 2-acetylpyridine precursor by 3-acetylpyridine. We [12,33,34] and Zhang and coworkers [35] have used this strategy to prepare a suite of 4′-(4-alkyloxyphenyl)-3,2′:6′,3″-terpyridines, including compound **1** with a 4-butoxyphenyl (Scheme 2). Reactions of 4-(butan-2-yloxy)benzaldehyde and 4-(*tert*-butoxy)benzaldehyde with two equivalents of 3-acetylpyridine in ethanol in the presence of KOH, followed by the addition of aqueous NH_3_, resulted in the formation of compounds *rac*-**2** and **4** in 24.8 and 20.5% yields, respectively (Scheme 4). We noted that *rac*-**2** was more soluble in EtOH than **1** [33] or **4**, and hence *rac*-**2** was recrystallized from MeOH/H_2_O rather than EtOH. The base peak in the ESI mass spectra of *rac*-**2** and **4** (Figures S1 and S2 in the Supplementary Materials) was observed at *m/z* 382.19 and 382.17, respectively, and was assigned to the [M + H]^+^ ion. The ^1^H and ^13^C{^1^H} NMR spectra of *rac*-**2** and **4** exhibited the characteristic spectroscopic signatures of 4′-(4-alkyloxyphenyl)-3,2′:6′,3″-terpyridines, and were assigned by 2D methods (Figures S3–S10 in the Supplementary Materials). The aromatic regions of the ^1^H NMR spectra (Figure 2a,b) were similar. However, when we attempted to prepare compound **3** from 4-(2-methylpropoxy)benzaldehyde and 3-acetylpyridine under comparable conditions, as for the syntheses of **2** and **4**, the colorless solid that was isolated exhibited the ^1^H and ^13^C{^1^H} NMR spectra shown in Figure 2c, Figures S11 and S12 in the Supplementary Materials. The appearance of the spectra was consistent with the formation of the cyclic product **3a** shown at the top of Scheme 4, and is analogous to the product formed during attempts to prepare 4′-(4-propoxyphenyl)-3,2′:6′,3″-terpyridine by the one-pot Wang and Hanan route [9]. The reasons for the failure of this procedure in the case of the propoxy and 2-methylpropoxy substituents remain unclear. Compound **3a** was fully characterized, and HMQC and HMBC spectra are displayed in Figures S13 and S14 in the Supplementary Materials. Figure S15 in the Supplementary Materials shows the ESI mass spectrum of **3a** with the base peak at *m/z* 684.28 corresponding to [M + H]^+^. Scheme 4 (bottom right) summarizes our alternative approach to compound **3**, which involved the reaction of 4′-(4-hydroxyphenyl)-3,2′:6′,3″-terpyridine with 1-bromo-2-methylpropane in basic conditions (Scheme 4), resulted in the formation of compound **3** in 19.2% yield after purification. The ESI mass spectrum (Figure S16 in the Supplementary Materials) and the ^1^H and ^13^C{^1^H} NMR spectra (Figure 2d, Figures S17–S20 in the Supplementary Materials) were consistent with the formation of **3**.

**Figure 3:**
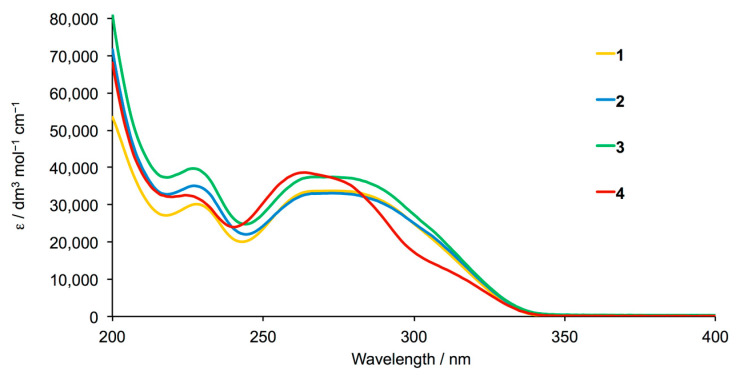
Solution absorption spectra of the isomeric compounds **1** (MeCN, 3.3 × 10^–5^ mol dm^–3^), *rac*-**2**, **3** and **4** (MeCN, 2.0 × 10^–5^ mol dm^–3^).

The solid-state IR spectra of the ligands (Figures S21–S23 in the Supplementary Materials) are comparable. Using **3** as an example, characteristic absorptions appear at 2955 and 2871 cm^–1^ in the C–H stretching region, as well as at 1604, 1513, 1241, 803 and 698 cm^–1^. The solution absorption spectra of *rac*-**2**, **3** and **4** are compared with that of **1** in Figure 3. The broad absorptions in the UV region arise mainly from π*←π transitions, and absorption maxima (Table 1) are similar.

### 3.2. Coordination Networks with Co(NCS)_2_

**Figure 4:**
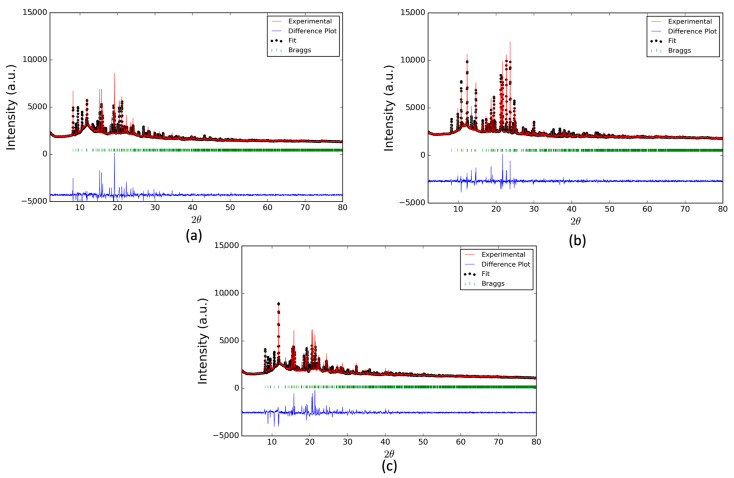
Laboratory powder X-ray diffraction (CuKα radiation) patterns (red lines) for (**a**) [{Co(*rac*-**2**)_2_(NCS)_2_}·CHCl_3_]*_n_*, (**b**) [Co(**3**)_2_(NCS)_2_]*_n_* and (**c**) [{Co(**4**)_2_(NCS)_2_}·CHCl_3_]*_n_* at room temperature, compared to the fitted patterns from the single-crystal X-ray diffraction data (black dots). The green lines denote the Bragg peak positions, and blue trace shows the difference between experimental and calculated points.

Crystals of coordination networks formed between Co(NCS)_2_ and ligands *rac*-**2**, **3** and **4** were grown by layering a methanol solution of Co(NCS)_2_ over a chloroform solution of the ligand. A suitable crystal of each product was selected for single-crystal X-ray diffraction, and the remaining pink blocks were collected, dried and analyzed by IR spectroscopy and PXRD. The FT-IR spectra of the crystals are displayed in Figures S24–S26 in the Supplementary Materials. The strong absorption band at 2069 cm^–1^ (compound with ligand **2**), 2061 cm^–1^ (with **3**) and 2074 cm^–1^ (with **4**) is assigned to the coordinated [NCS]^–^ ligands. Single crystal structure determinations revealed formulations of [{Co(*rac*-**2**)_2_(NCS)_2_}·CHCl_3_]*_n_*, [Co(**3**)_2_(NCS)_2_]*_n_* and [{Co(**4**)_2_(NCS)_2_}·CHCl_3_]*_n_*, and PXRD (Figure 4) confirmed that the single crystal structures were representative of the bulk samples. Every peak present in the experimental plot finds a correspondence in the fit, and the differences in the intensities are mostly due to differences in the preferred orientations in the powdered bulk sample.

**Figure 5:**
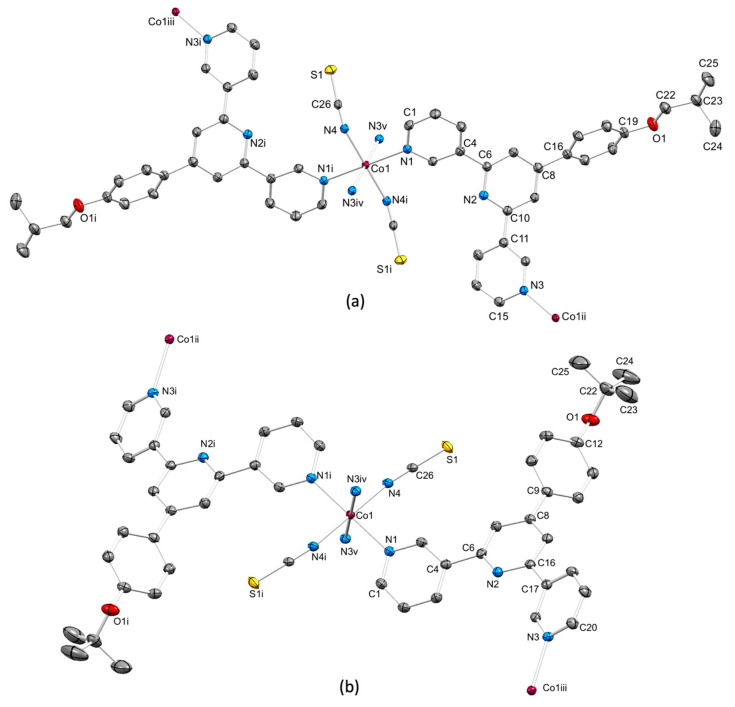
ORTEP representations of the coordination sphere of atom Co1 (with symmetry generated Co centers) in (**a**) [Co(**3**)_2_(NCS)_2_]*_n_* (symmetry codes: i = 2–*x*, 2–*y*, 1–*z*; ii = ^3^/_2_–*x*, –^1^/_2_ + *y*, ^1^/_2_–*z*; iii = ^5^/_2_–*x*, ^1^/_2_ + *y*, ^3^/_2_–*z*; iv = ^3^/_2_–*x*, ^1^/_2_ + *y*, ^1^/_2_–*z*; v = ^1^/_2_ + *x*, ^3^/_2_–*y*, ^1^/_2_ + *z*) and (**b**) [{Co(**4**)_2_(NCS)_2_}·CHCl_3_]*_n_* (symmetry codes: i = 1–*x*, 1–*y*, –*z*; ii = ^1^/_2_–*x*, ^1^/_2_ + *y*, –^1^/_2_–*z*; iii = ^3^/_2_–*x*, –^1^/_2_ + *y*, ^1^/_2_–*z*; iv = –^1^/_2_ + *x*, ^1^/_2_–*y*, –^1^/_2_ + *z*; v = ^3^/_2_–*x*, ^1^/_2_ + *y*, ^1^/_2_–*z*). Ellipsoids are plotted at 40% probability level and H atoms are omitted.

Each of [{Co(*rac*-**2**)_2_(NCS)_2_}·CHCl_3_]*_n_*, [Co(**3**)_2_(NCS)_2_]*_n_* and [{Co(**4**)_2_(NCS)_2_}·CHCl_3_]*_n_* crystallize in the monoclinic space group *P*2_1_/*n*. This is in contrast to [{Co(**1**)_2_(NCS)_2_}·4CHCl_3_]*_n_* (Figure 1), which crystallizes in the tetragonal space group *P–*42_1_*c* [6]. ORTEP representations of the repeat units in [Co(**3**)_2_(NCS)_2_]*_n_* and [{Co(**4**)_2_(NCS)_2_}·CHCl_3_]*_n_* are shown in Figure 5, and that of [{Co(*rac*-**2**)_2_(NCS)_2_}·CHCl_3_]*_n_* is displayed in Figure S27 in the Supplementary Materials. The Co–N bond lengths (Table 2) are unremarkable, and the N_tpy_-Co-N_tpy_ and N_NCS_-Co-N_CNS_ bond angles in Table 2 follow from the location of atom Co1 in each structure on an inversion center. In each structure, the 3,2′:6′,3″-tpy unit adopts conformation **II** (Scheme 1), which differs from the ligand conformation **I** observed in [{Co(**1**)_2_(NCS)_2_}·4CHCl_3_]*_n_* [6].

**Figure 6:**
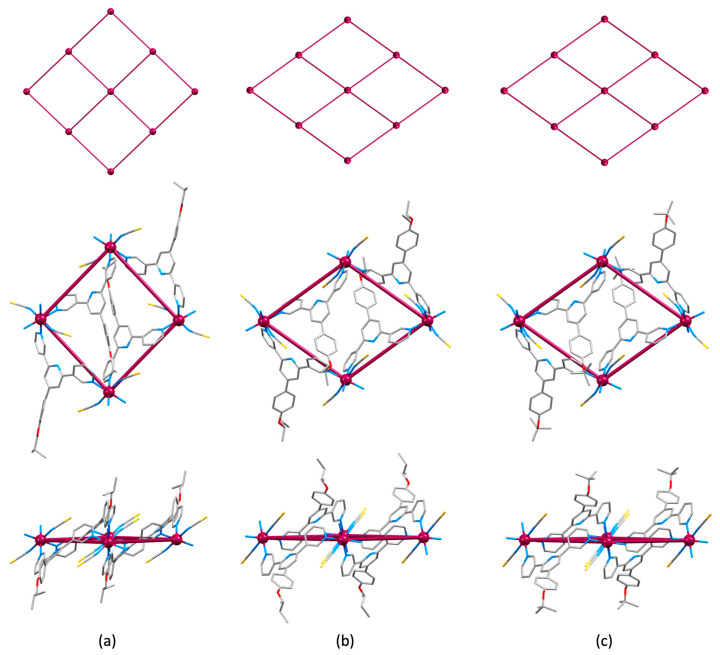
Comparison of the (4,4) nets in (**a**) [Co(**3**)_2_(NCS)_2_]*_n_*, (**b**) [{Co(*rac*-**2**)_2_(NCS)_2_}·CHCl_3_]*_n_*, and (**c**) [{Co(**4**)_2_(NCS)_2_}·CHCl_3_]*_n_* (top, middle and bottom diagrams for each compound). The Co...Co vectors are drawn to emphasize the (4,4) net.

**Scheme 5:**
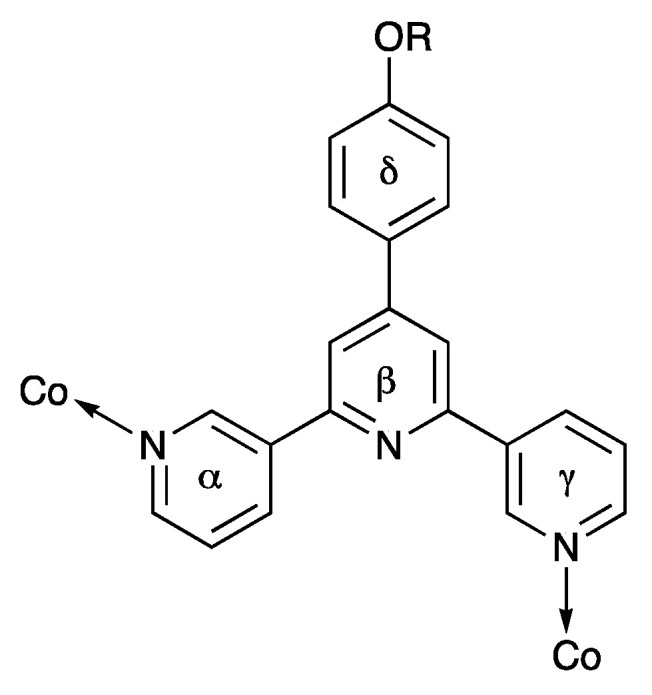
In [{Co(*rac*-**2**)_2_(NCS)_2_}·CHCl_3_]*_n_*, [Co(**3**)_2_(NCS)_2_]*_n_* and [{Co(**4**)_2_(NCS)_2_}·CHCl_3_]*_n_*, the ligand adopts conformation **II** (Scheme 1) and the Co atom is on an inversion center. Pyridine rings α and γ are therefore topologically distinct.

**Figure 7:**
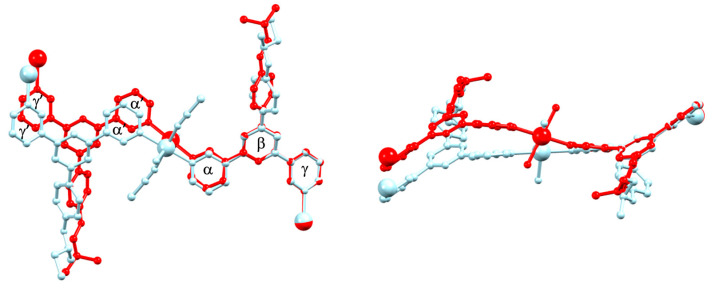
Overlay of parts of the structures of [Co(**3**)_2_(NCS)_2_]*_n_* (red) and [{Co(*rac*-**2**)_2_(NCS)_2_}·CHCl_3_]*_n_* (pale blue). Thiocyanato ligands and H atoms are omitted. See text for details.

2-Dimensional networks assemble for each of [{Co(*rac*-**2**)_2_(NCS)_2_}·CHCl_3_]*_n_*, [Co(**3**)_2_(NCS)_2_]*_n_* and [{Co(**4**)_2_(NCS)_2_}·CHCl_3_]*_n_*. The topology of each net is the same (Figure 6a–c, top), with *trans*-{Co(NCS)_2_N_4_} 4-connecting nodes, and 3,2′:6′,3″-tpy ligands bridging between adjacent of Co centers (Figure 6a–c, middle and bottom). Working sequentially around the edges of a rhombus in each network, the ligands are arranged in a down/down/up/up pattern, which contrasts with the cone-like arrangement found in [{Co(**1**)_2_(NCS)_2_}·4CHCl_3_]*n* (Figure 1b). We note that the crystallographic symmetry dictates that both the (*R*)-**2** and (*S*)-**2** enantiomers are present is a single 2D-net of [{Co(*rac*-**2**)_2_(NCS)_2_}·CHCl_3_]*_n_*, rendering the net heterochiral. Figure 6b,c illustrate that the structures of the networks in [{Co(*rac*-**2**)_2_(NCS)_2_}·CHCl_3_]*_n_* and [{Co(**4**)_2_(NCS)_2_}·CHCl_3_]*_n_* are essentially the same, and this is confirmed by inspection of the overlays depicted in Figure S28 in the Supporting Materials. Thus, a change in the steric effects from butan-2-yloxy to *tert*-butoxy has negligible impact on the coordination network. However, the network deforms on going to ligand **3** with the 2-methylpropoxy substituent (Figure 6a), and the cause can be traced to a small conformational change. The angles between the planes of pairs of bonded arene rings in each coordinated ligand are summarized in Table 3. Because the ligand is in conformation **II**, the outer pyridine rings in the coordinated 3,2′:6′,3″-tpy are distinct from each other (Scheme 5). The angles between rings β and γ are similar in all three structures, but the angle between rings α and β is larger in [Co(**3**)_2_(NCS)_2_]*_n_* than in [{Co(*rac*-**2**)_2_(NCS)_2_}·CHCl_3_]*_n_* and [{Co(**4**)_2_(NCS)_2_}·CHCl_3_]*_n_* (Table 3). This has a significant impact on the propagation of the 2D-structure, as can be seen from Figure 7, which displays an overlay of parts of the structures of [Co(**3**)_2_(NCS)_2_]*_n_* and [{Co(*rac*-**2**)_2_(NCS)_2_}·CHCl_3_]*_n_*. The Co atoms and rings C that are labeled in Figure 7 are perfectly overlaid. The larger twist angle between rings α and β in [Co(**3**)_2_(NCS)_2_]*_n_* compared to [{Co(*rac*-**2**)_2_(NCS)_2_}·CHCl_3_]*_n_* leads to a redirecting of the Co...Co vector (Figure 7), and, consequently, a deformation of the (4,4) network. This highlights the manner in which small conformational changes within the 3,2′:6′,3″-tpy domain can accommodate changes in the spatial properties of substituents, without leading to significant topological modifications. Note that from crystal symmetry, it follows that *trans*-arrangements must be (ring α)–Co–(ring α) or (ring γ)–Co–(ring γ), and that every Co atom is bonded to two α and two γ rings.

## 4. Conclusions

We have prepared and characterized three isomeric 3,2′:6′,3″-tpy ligands, *rac*-**2**, **3** and **4**, which possess *rac*-4′-(4-butan-2-yloxyphenyl), 4′-(2-methylpropoxyphenyl) and 4′-(*tert*-butoxyphenyl) substituents, respectively. Reactions of these ligands with Co(NCS)_2_ under conditions of crystal growth at room temperature resulted in the formation of [{Co(*rac*-**2**)_2_(NCS)_2_}·CHCl_3_]*_n_*, [Co(**3**)_2_(NCS)_2_]*_n_* and [{Co(**4**)_2_(NCS)_2_}·CHCl_3_]*_n_*, which possess (4,4) networks, with the Co centers acting as 4-connecting nodes. The down/down/up/up arrangement of four 3,2′:6′,3″-tpy linkers around a rhombus in each net contrasts with the cone-like arrangement of 4′-(butoxyphenyl)-3,2′:6′,3″-tpy (**1**) ligands in the previously reported (4,4) net in [{Co(**1**)_2_(NCS)_2_}·4CHCl_3_]*n* [6]. In the latter, a cone of four extended butoxy chains is accommodated with a similar cone in the next sheet. Whereas the switch from the ligand with a 4′-(butoxyphenyl) substituent to the three isomers *rac*-**2**, **3** and **4** with branched chains causes significant structural perturbation (Figure 1 compared to Figure 6), changes in the spatial properties of the branched substituents are accommodated with subtle conformational changes in the 3,2′:6′,3″-tpy domain.

## Figures and Tables

**Table 1:** Solution absorption maxima (MeCN, 2.0 × 10^−5^ mol dm^−3^) for compounds *rac*-**2**, **3** and **4**.

Compound	λ/nm (ε/dm^3^ mol^–1^ cm^–1^)
*rac*-**2**	227 (35,100), 273 (33,100)
**3**	227 (39,700), 269 (37,450)
**4**	224 (32,550), 264 (38,600), 315 sh (10,800)

**Table 2:** Selected bond lengths and angles ^1^ in [{Co(*rac*-**2**)_2_(NCS)_2_}·CHCl_3_]*_n_*, [Co(**3**)_2_(NCS)_2_]*_n_* and [{Co(**4**)_2_(NCS)_2_}·CHCl_3_]*_n_*.

Compound	Co–N_tpy_/Å	Co–N_NCS_/Å	N_tpy_–Co–N_tpy_^1^/deg	N_NCS_–Co–N_NCS_ /deg
[{Co(*rac*-**2**)_2_(NCS)_2_}·CHCl_3_]*_n_*	2.185(3), 2.211(3)	2.079(3)	180, 180	180
[Co(**3**)_2_(NCS)_2_]*_n_*	2.217(3), 2.219(3)	2.072(3)	180, 180	180
[{Co(**4**)_2_(NCS)_2_}·CHCl_3_]*_n_*	2.205(3), 2.230(3)	2.066(3)	180, 180	180

^1^ Only the *trans* angles are given.

**Table 3:** Angles between the arene ring-planes in [{Co(*rac*-**2**)_2_(NCS)_2_}·CHCl_3_]*_n_*, [Co(**3**)_2_(NCS)_2_]*_n_* and [{Co(**4**)_2_(NCS)_2_}·CHCl_3_]*_n_*. The pyridine rings are labelled as defined in Scheme 5.

Compound	Ring α/Ring β	Ring β/Ring γ	Ring β/Ring δ
[{Co(*rac*-**2**)_2_(NCS)_2_}·CHCl_3_]*_n_*	30.6	32.7	55.7
[Co(**3**)_2_(NCS)_2_]*_n_*	37.7	27.9	48.1
[{Co(**4**)_2_(NCS)_2_}·CHCl_3_]*_n_*	28.0	28.9	48.3

## Supplementary Materials

The following are available online at https://www.mdpi.com/2073-4360/12/8/1823/s1, Figures S1–S23: Electrospray mass spectra, NMR spectra and solid-state IR spectra of compounds *rac*-**2**, **3** and **4**; Figures S24–S26: solid-state IR spectra of the coordination networks; Figure S27: ORTEP representation of the repeat unit in [{Co(**2**)_2_(NCS)_2_}·CHCl_3_]*_n_*; Figure S28: Overlays of the (4,4) nets in [{Co(*rac*-**2**)_2_(NCS)_2_}·CHCl_3_]*_n_* and [{Co(**4**)_2_(NCS)_2_}·CHCl_3_]*_n_*.
