# Supplementary material for: Straight Versus Branched Chain Substituents in 4′-(Butoxyphenyl)-3,2′:6′,3″-terpyridines: Effects on (4,4) Coordination Network Assemblies
Source: Polymers (Basel). 2020 Aug 14;12(8):1823. doi: 10.3390/polym12081823 (PMC7465904; doi:10.3390/polym12081823)
Supplement: Supplementary file 1 [file polymers-12-01823-s001.pdf]

Supplementary Materials:

Straight versus branched chain substituents in 4'-(butoxyphenyl)-3,2':6',3"-terpyridines: Effects on (4,4) coordination network assemblies

Dalila Rocco [1], Alessandro Prescimone [1], Edwin C. Constable [1] and Catherine E. Housecroft [1]*

[1] Department of Chemistry, University of Basel, BPR 1096, Mattenstrasse 24a, CH-4058 Basel, Switzerland; dalila.rocco@unibas.ch (D.R.); alessandro.prescimone@unibas.ch (A.P.); edwin.constable@unibas.ch (E.C.C.), catherine.housecroft@unibas.ch (C.E.H.)

* Correspondence: catherine.housecroft@unibas.ch; Tel: +41 61 207 1008

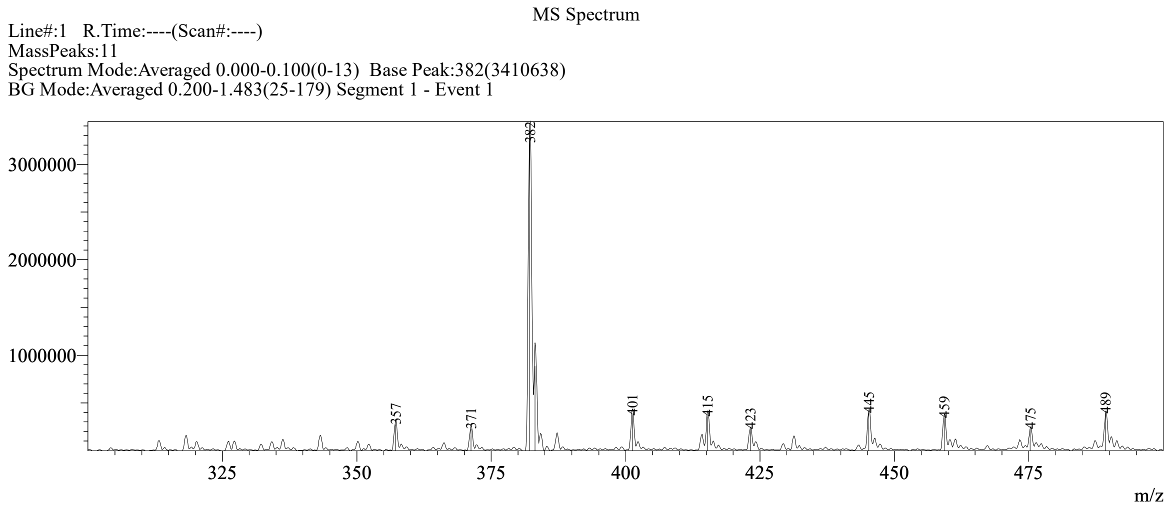

Figure. S1. Electrospray mass spectrum of *rac*-**2**.

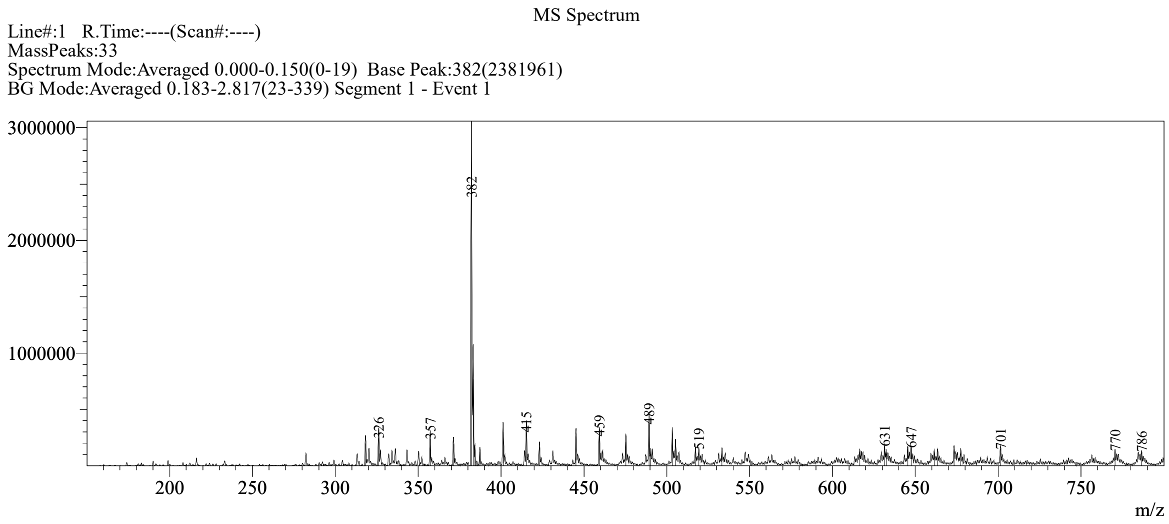

Figure. S2. Electrospray mass spectrum of **4**.

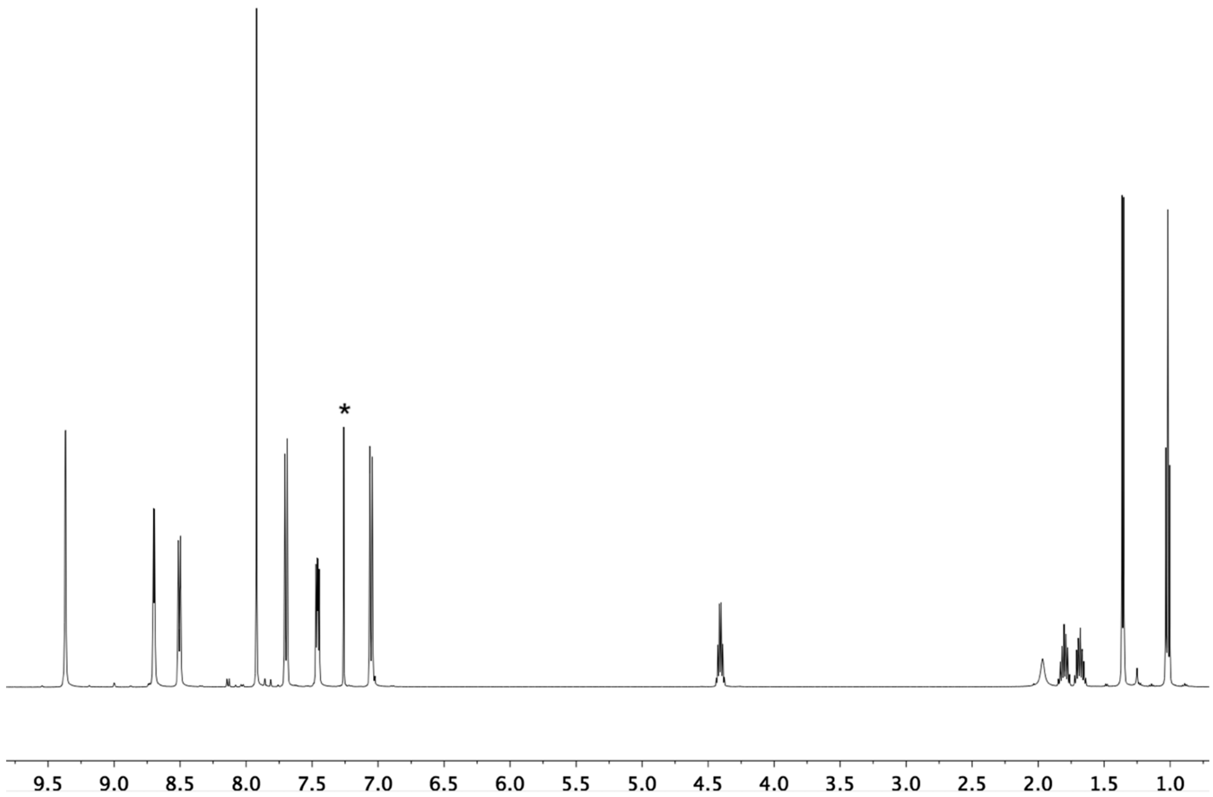

Figure. S3. ^{1}H NMR spectrum of compound *rac*-**2** (500 MHz, 298 K, $CDCl_3$). * = residual $CHCl_3$.

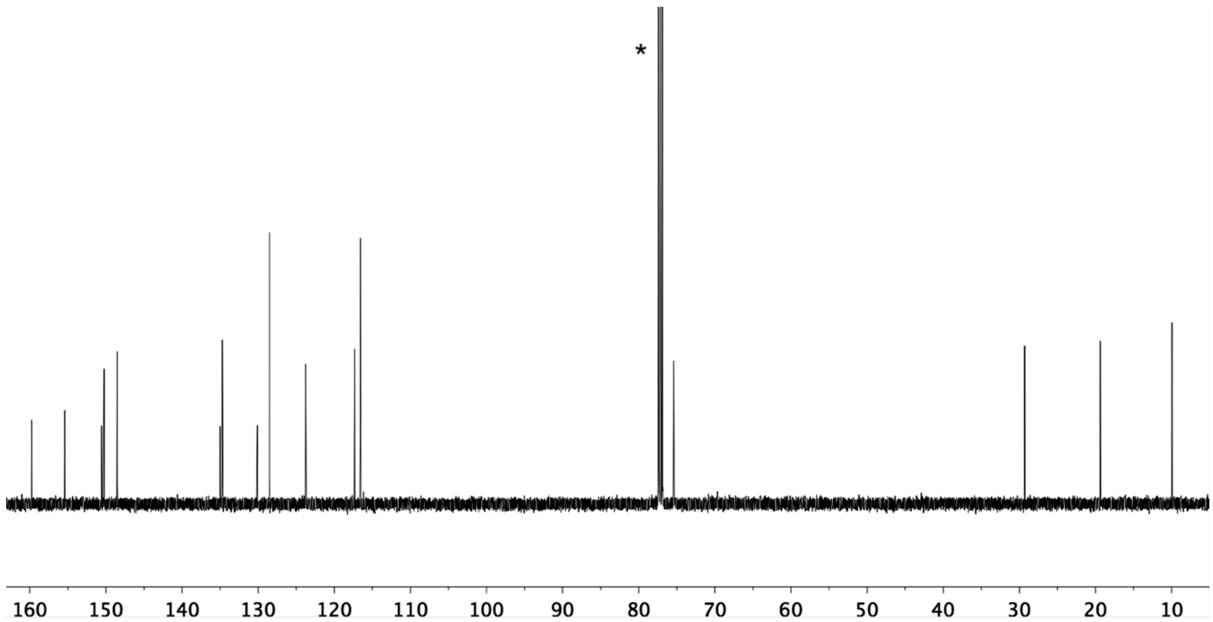

Figure. S4. $^{13}C\{^{1}H\}$ NMR spectrum of compound *rac*-**2** (126 MHz, 298 K, $CDCl_3$). * = $CDCl_3$.

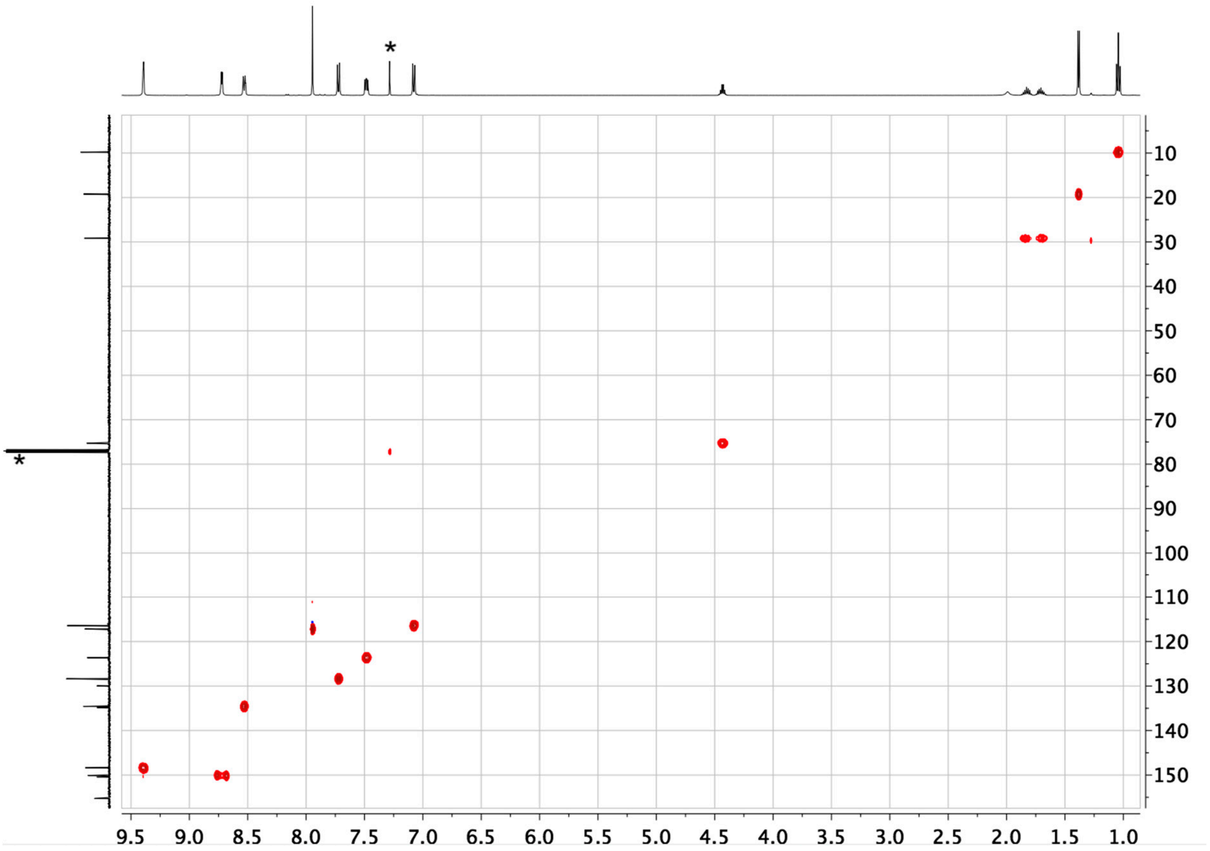

Figure. S5. HMQC spectrum of compound *rac*-**2** (^{1}H 500 MHz, ^{13}C 126 MHz, 298 K, $CDCl_3$). * = residual $CHCl_3$ or $CDCl_3$.

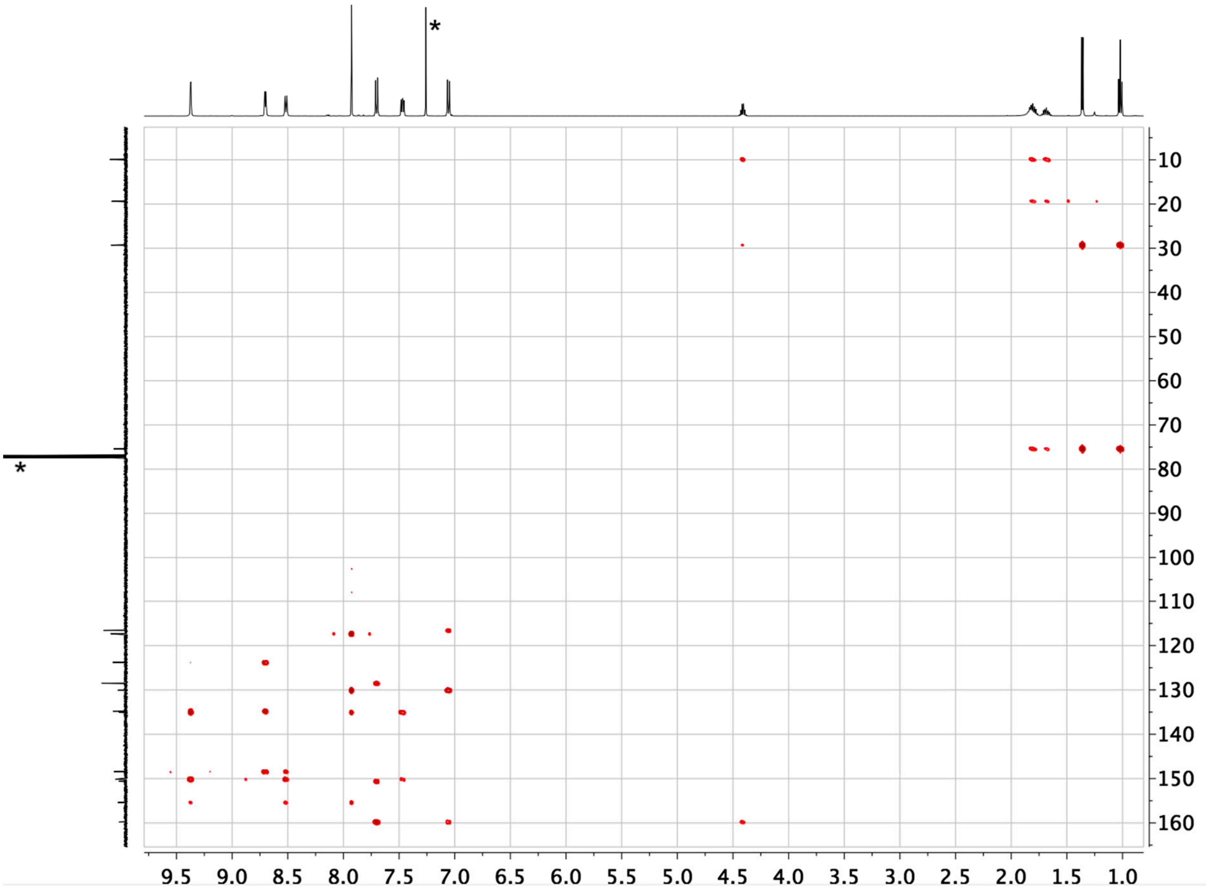

Figure. S6. HMBC spectrum of compound *rac*-**2** (^{1}H 500 MHz, ^{13}C 126 MHz, 298 K, $CDCl_3$). * = residual $CHCl_3$ or $CDCl_3$.

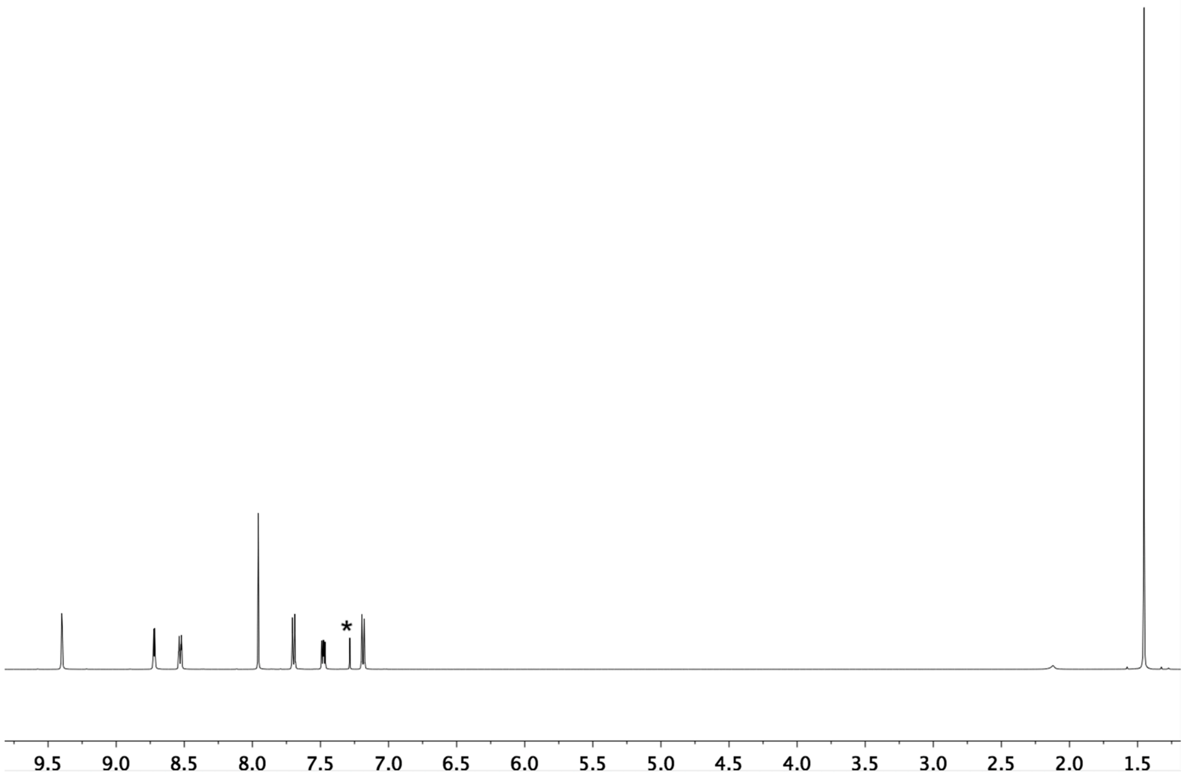

Figure. S7. ^{1}H NMR spectrum of compound **4** (500 MHz, 298 K, $CDCl_3$). * = residual $CHCl_3$.

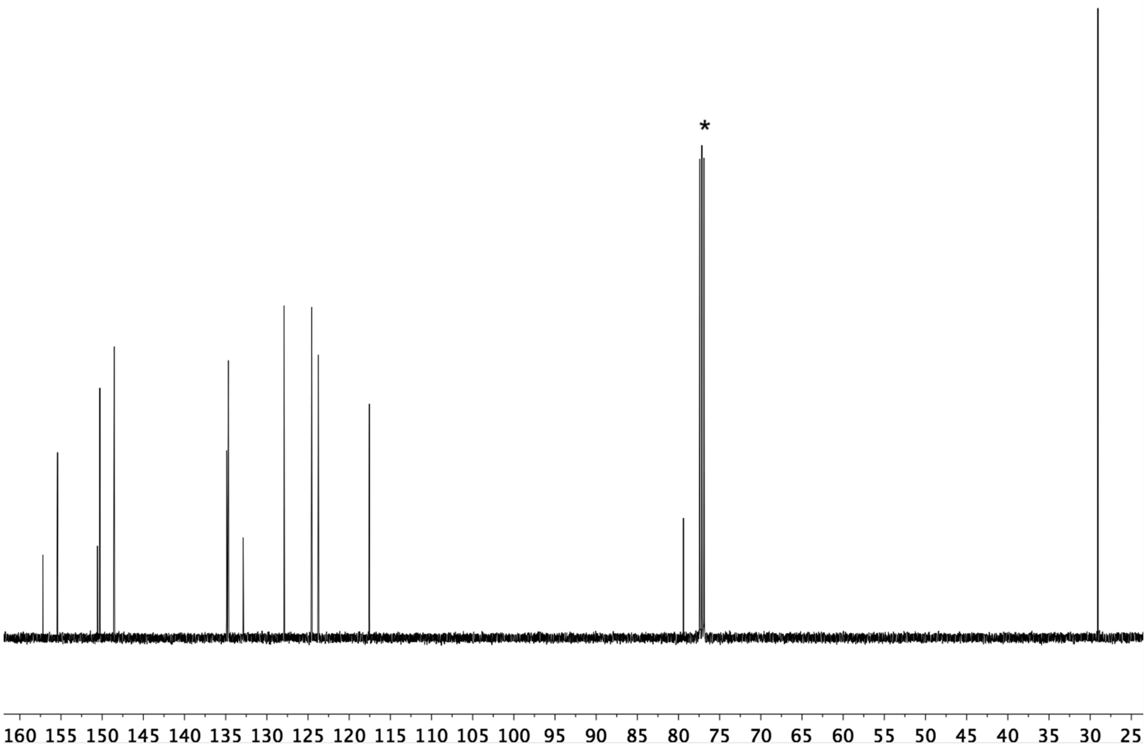

Figure. S8. $^{13}C\{^{1}H\}$ NMR spectrum of compound **4** (126 MHz, 298 K, $CDCl_3$). * = $CDCl_3$.

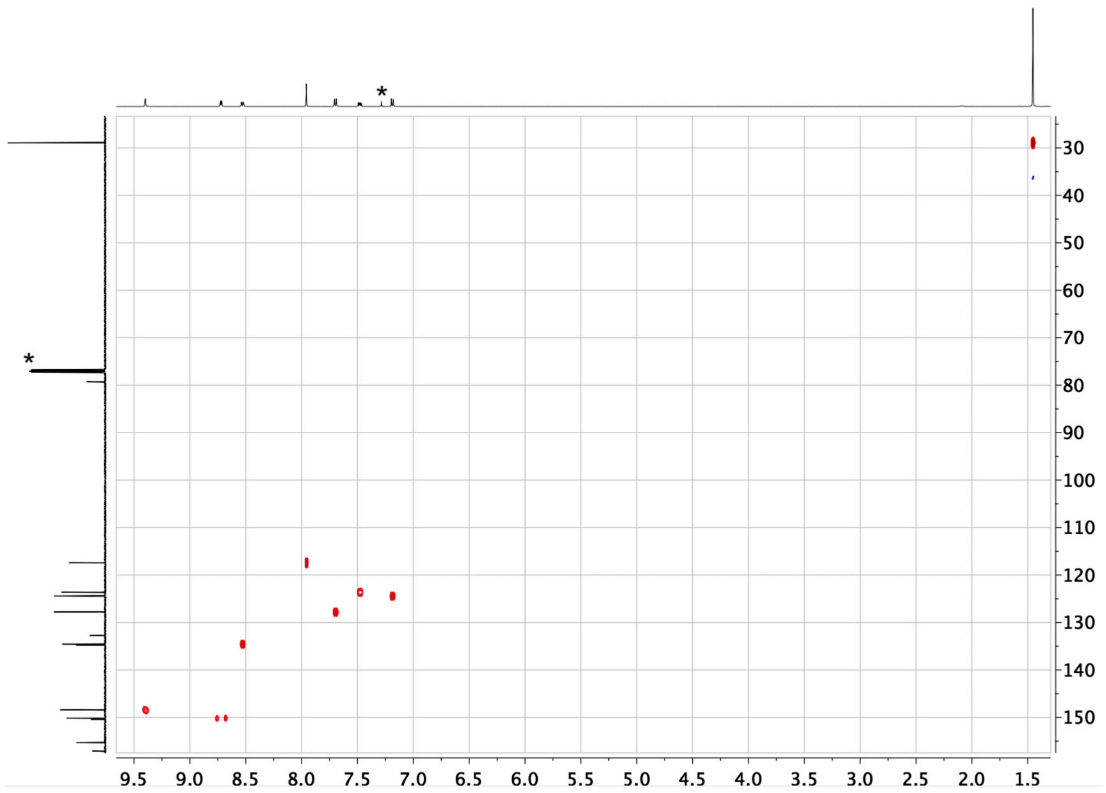

Figure. S9. HMQC spectrum of compound **4** (^{1}H 500 MHz, ^{13}C 126 MHz, 298 K, $CDCl_3$). * = residual $CHCl_3$ or $CDCl_3$.

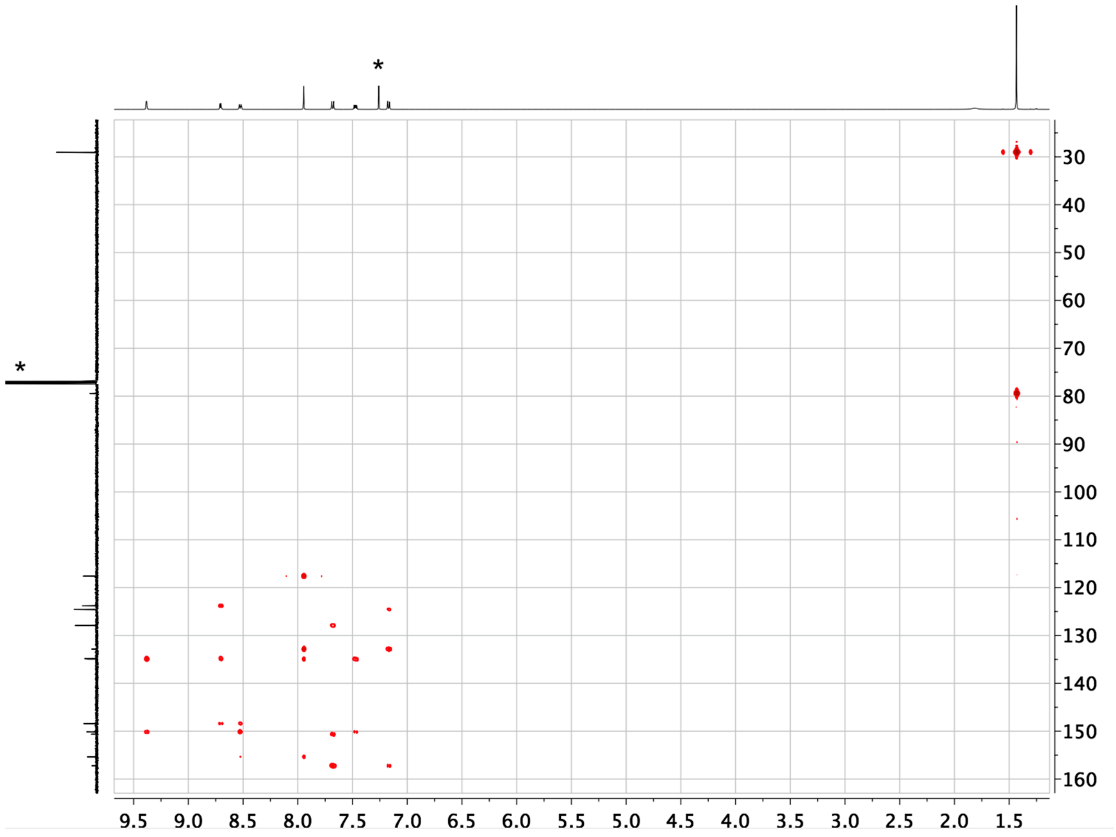

Figure. S10. HMBC spectrum of compound **4** (^{1}H 500 MHz, ^{13}C 126 MHz, 298 K, $CDCl_3$). * = residual $CHCl_3$ or $CDCl_3$.

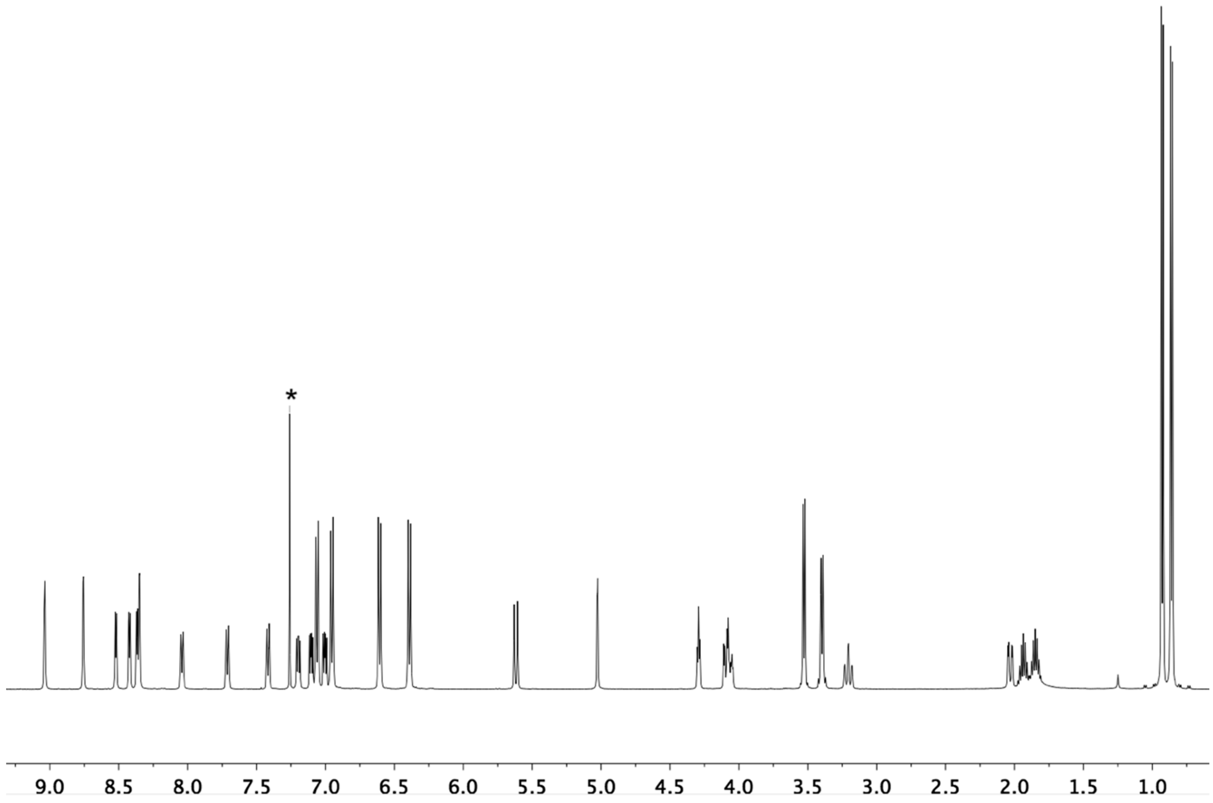

Figure. S11. ^{1}H NMR spectrum of compound **3a** (500 MHz, 298 K, $CDCl_3$). * = residual $CHCl_3$.

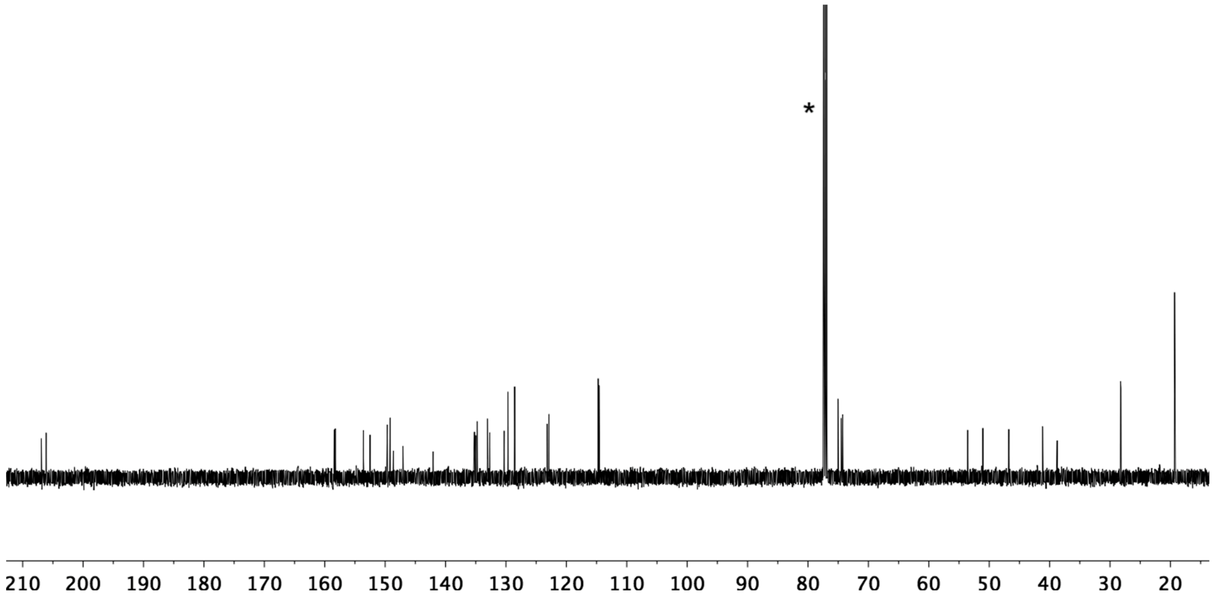

Figure. S12. $^{13}C\{^{1}H\}$ NMR spectrum of compound **3a** (126 MHz, 298 K, $CDCl_3$). * = $CDCl_3$.

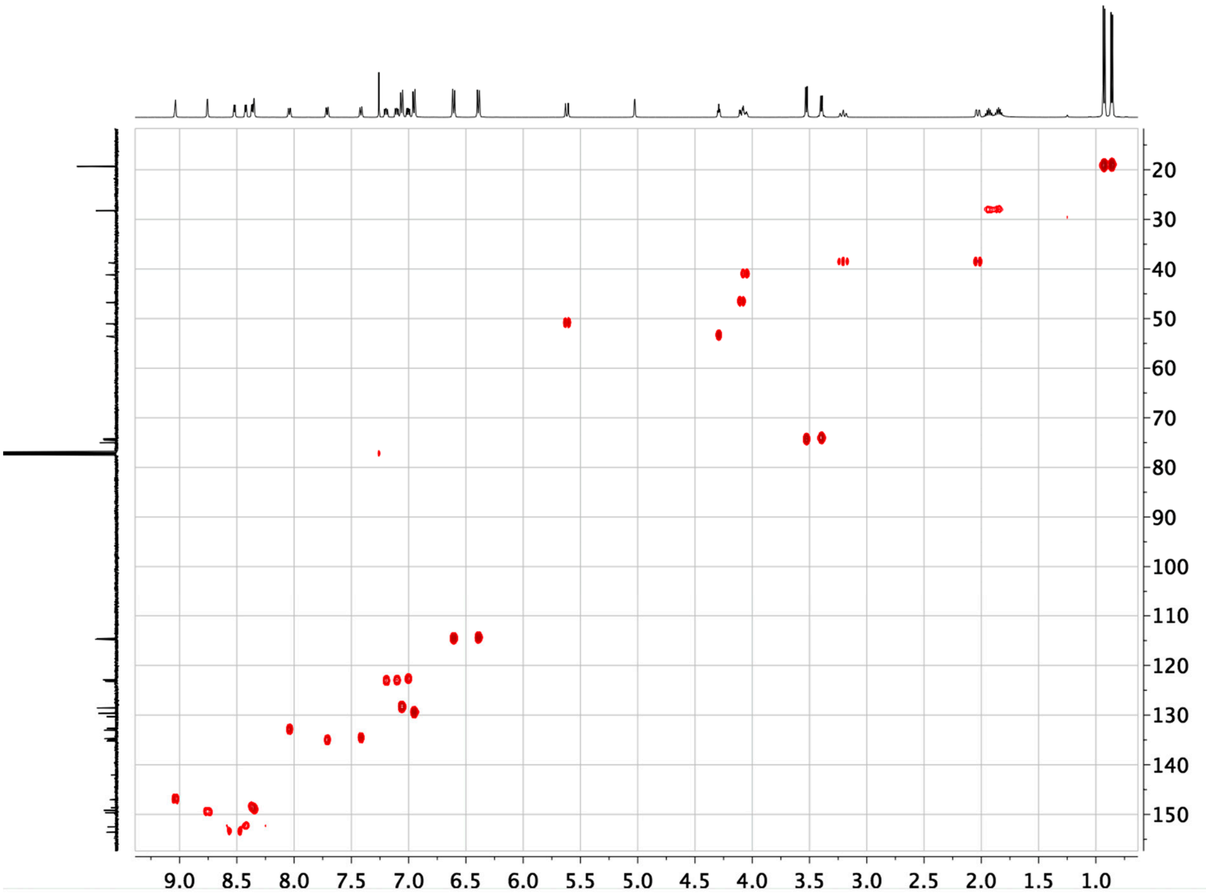

Figure. S13. HMQC spectrum of compound **3a** (^{1}H 500 MHz, ^{13}C 126 MHz, 298 K, $CDCl_3$). * = residual $CHCl_3$ or $CDCl_3$.

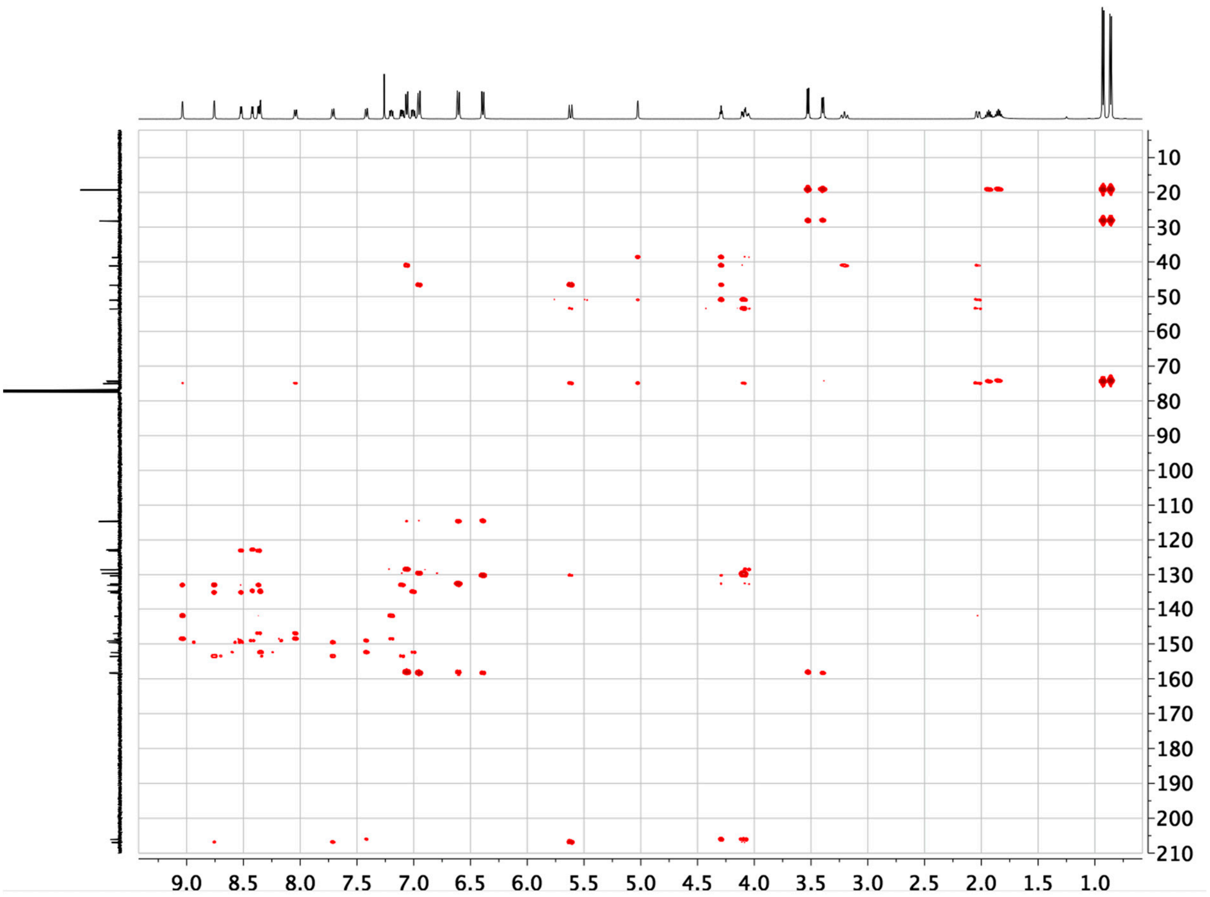

Figure. S14. HMBC spectrum of compound **3a** (^{1}H 500 MHz, ^{13}C 126 MHz, 298 K, $CDCl_3$). * = residual $CHCl_3$ or $CDCl_3$.

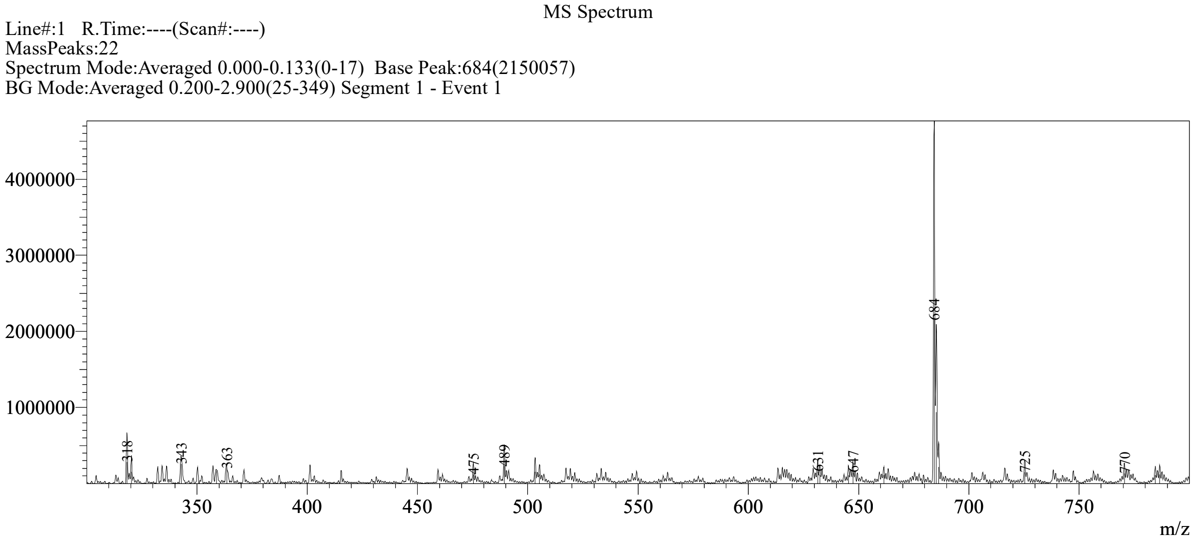

Figure. S15. Electrospray mass spectrum of compound **3a**.

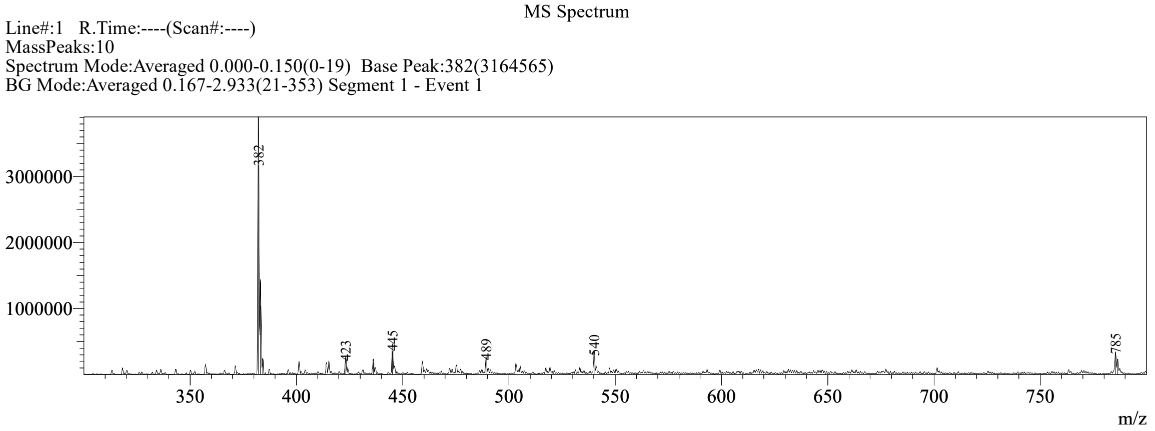

Figure. S16. Electrospray mass spectrum of compound **3**.

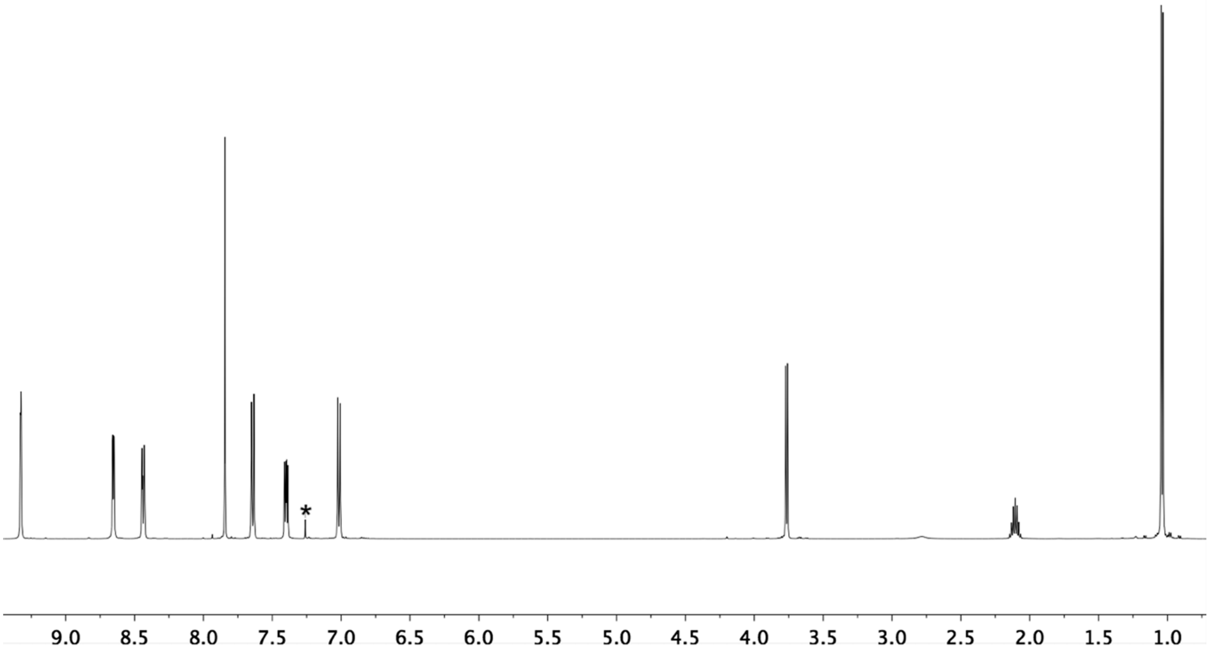

Figure. S17. ^{1}H NMR spectrum of compound **3** (500 MHz, 298 K, $CDCl_3$). * = residual $CHCl_3$.

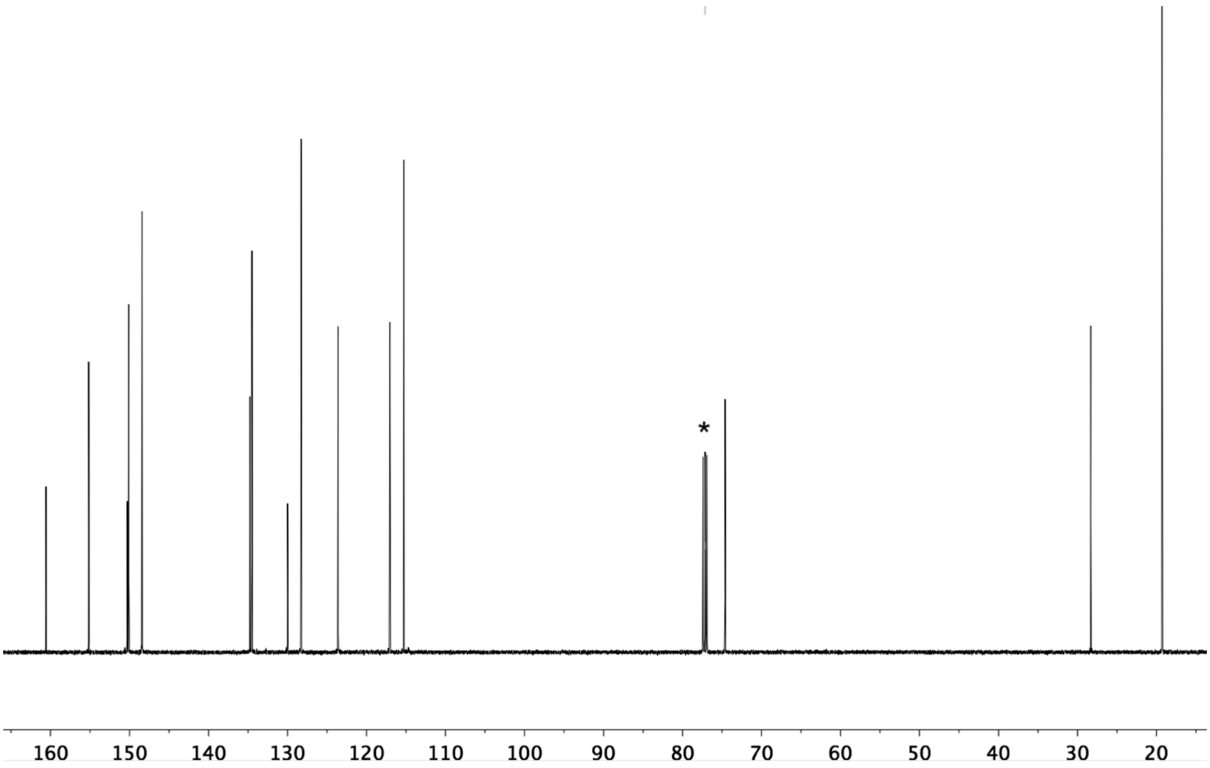

Figure. S18. $^{13}C\{^{1}H\}$ NMR spectrum of compound **3** (126 MHz, 298 K, $CDCl_3$). * = $CDCl_3$.

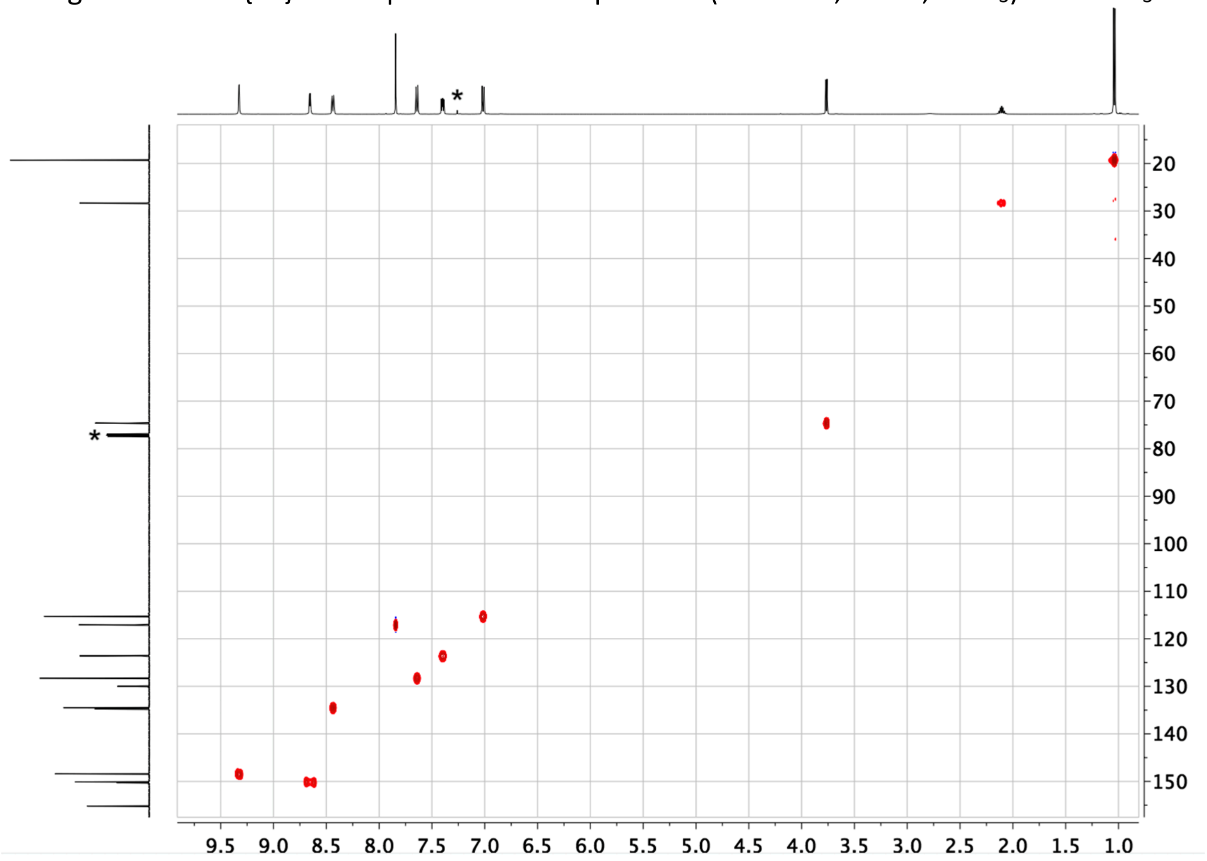

Figure. S19. HMQC spectrum of compound **3** (^{1}H 500 MHz, ^{13}C 126 MHz, 298 K, $CDCl_3$). * = residual $CHCl_3$ or $CDCl_3$.

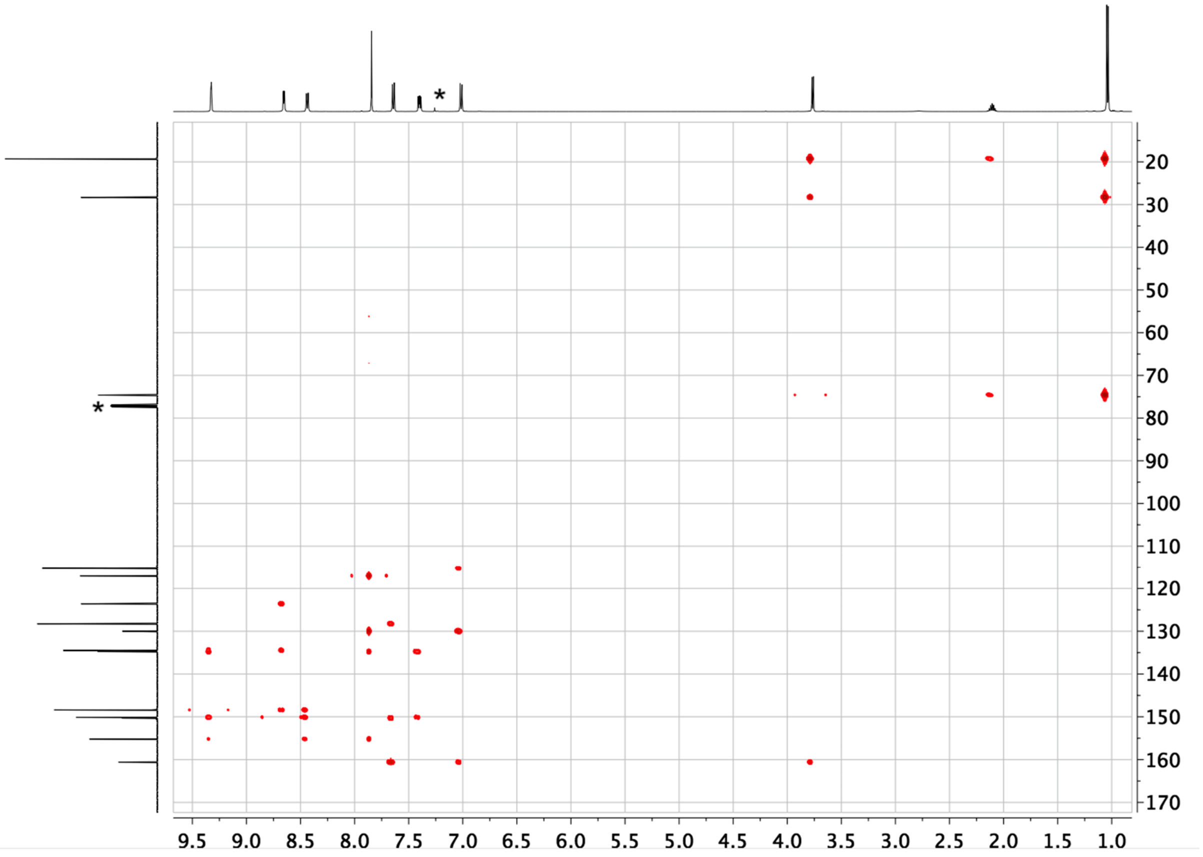

Figure. S20. HMBC spectrum of compound **3** (^{1}H 500 MHz, ^{13}C 126 MHz, 298 K, $CDCl_3$). * = residual $CHCl_3$ or $CDCl_3$.

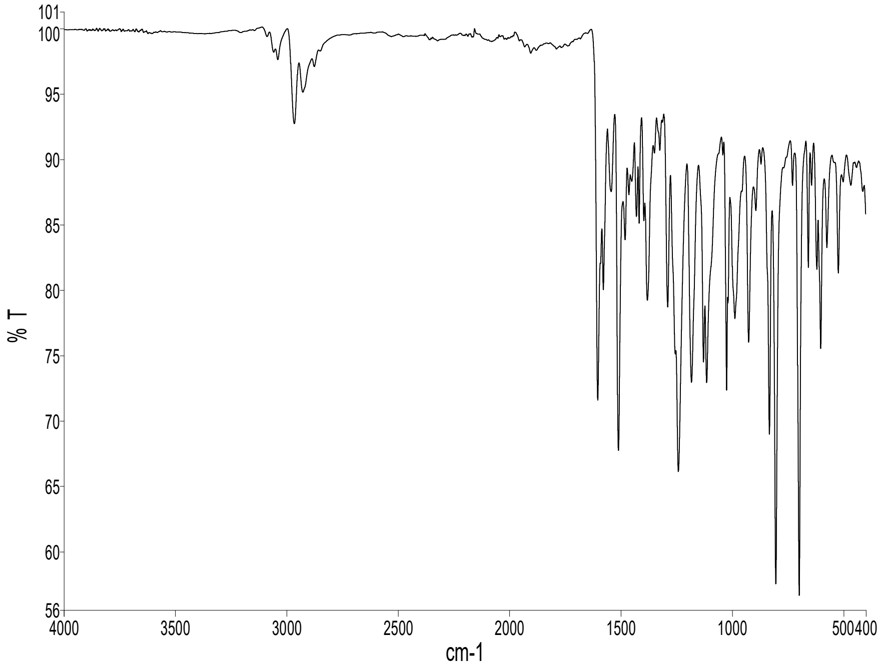

Figure. S21. The solid-state FT-IR spectrum of *rac*-**2**.

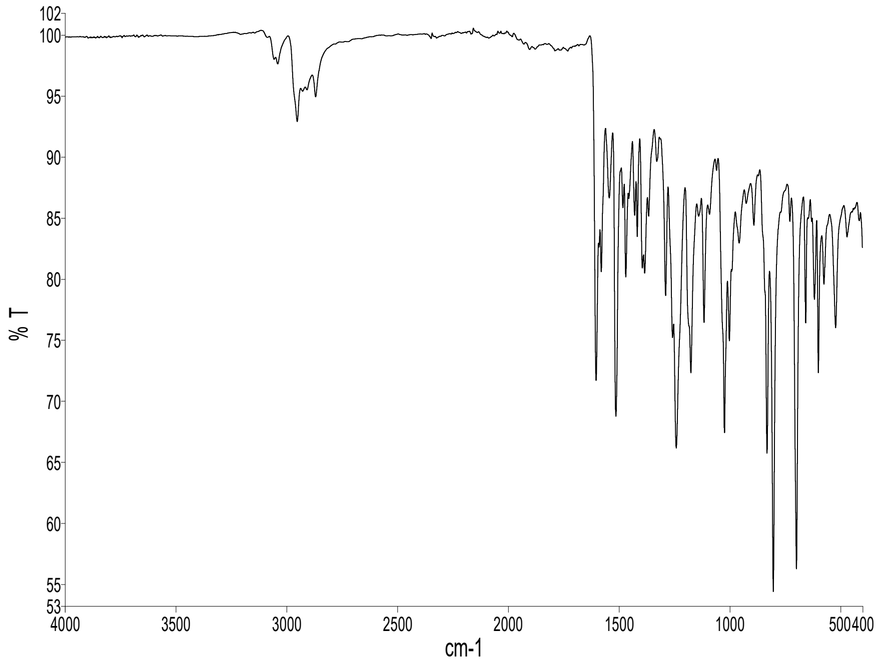

Figure. S22. The solid-state FT-IR spectrum of **3**.

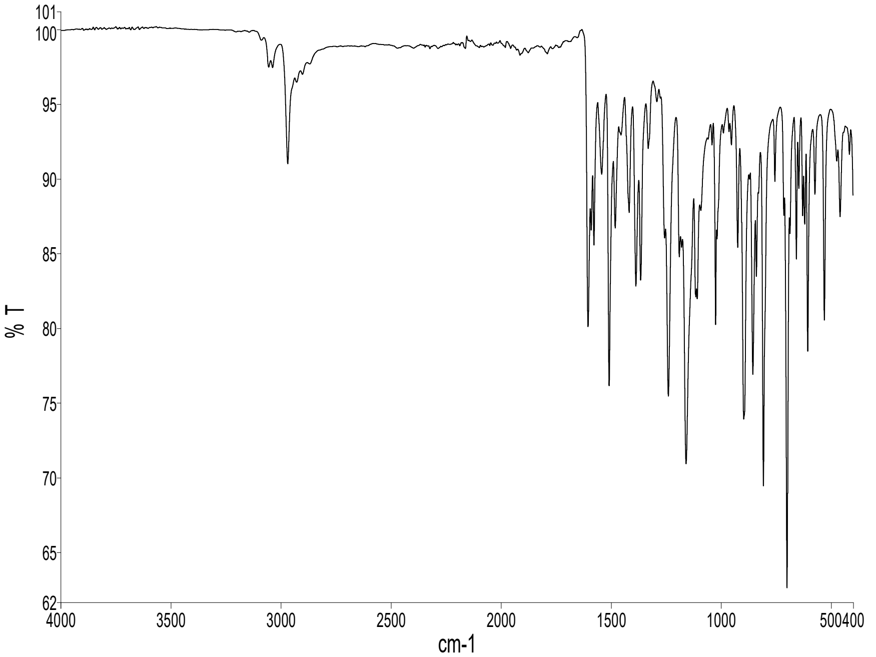

Figure. S23. The solid-state FT-IR spectrum of **4**.

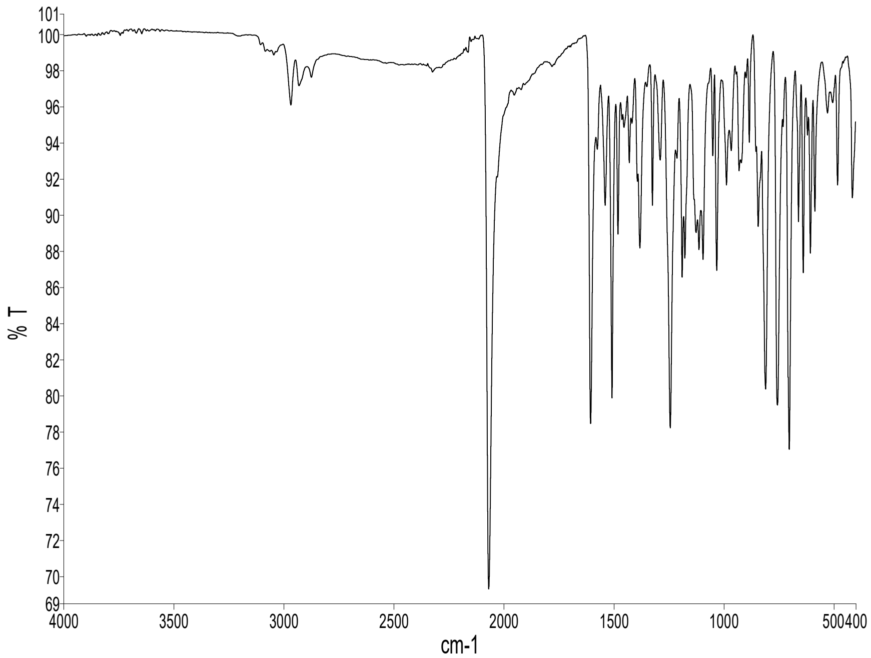

Figure. S24. The solid-state FT-IR spectrum of [{Co(*rac*-**2**)$_2$(NCS)$_2$}·$CHCl_3$]$_n$.

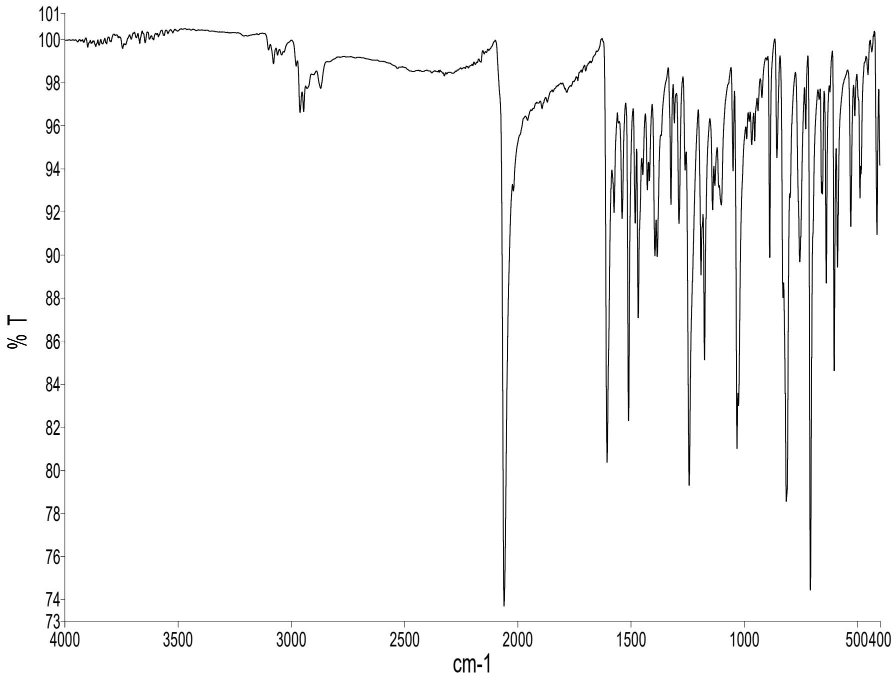

Figure. S25. The solid-state FT-IR spectrum of [{Co(**3**)$_2$(NCS)$_2$}]$_n$.

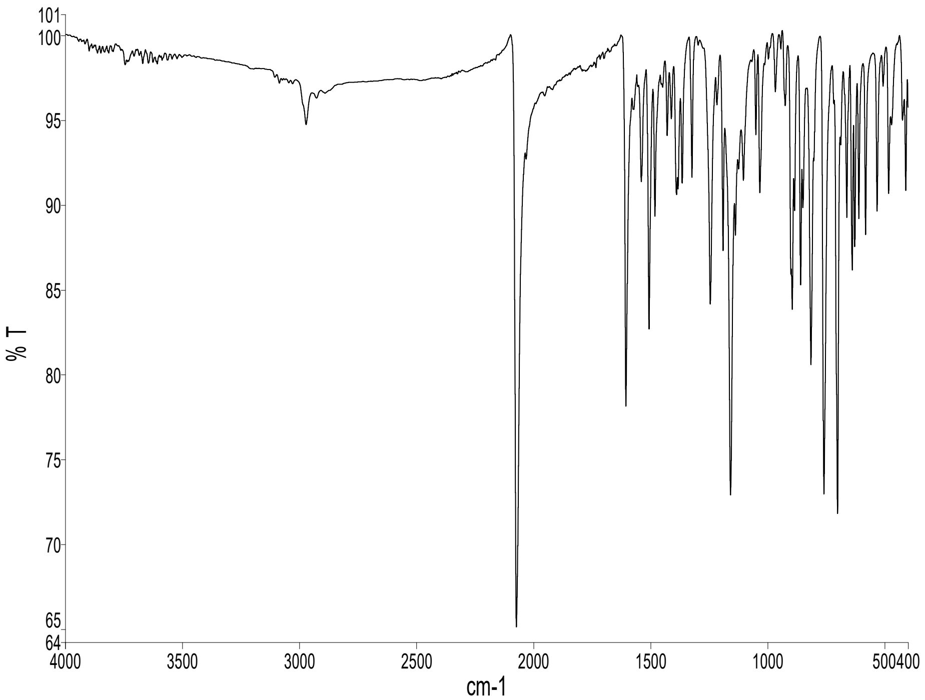

Figure. S26. The solid-state FT-IR spectrum of [{Co(**4**)$_2$(NCS)$_2$}·$CHCl_3$]$_n$.

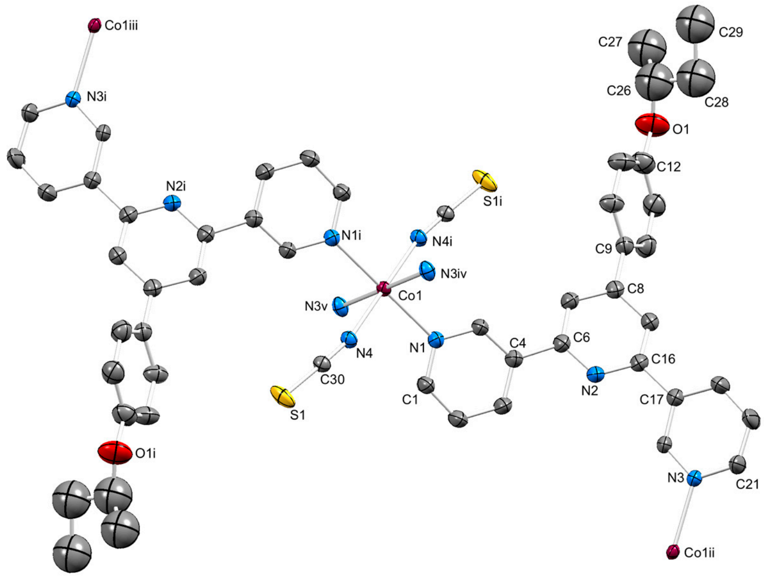

Figure. S27. ORTEP representation of the coordination sphere of atom Co1 (with symmetry generated Co centers) in [{Co(*rac*-**2**)$_2$(NCS)$_2$}·$CHCl_3$]$_n$ (symmetry codes: i = 1–*x*, 1–*y*, 1–*z*; ii = $^{1}/_{2}-x$, $-^{1}/_{2}+y$, $^{1}/_{2}-z$; iii = $^{3}/_{2}-x$, $^{1}/_{2}+y$, $^{3}/_{2}-z$; iv = $^{1}/_{2}+x$, $^{1}/_{2}-y$, $^{1}/_{2}+z$; v = $^{1}/_{2}-x$, $^{1}/_{2}+y$, $^{1}/_{2}-z$).

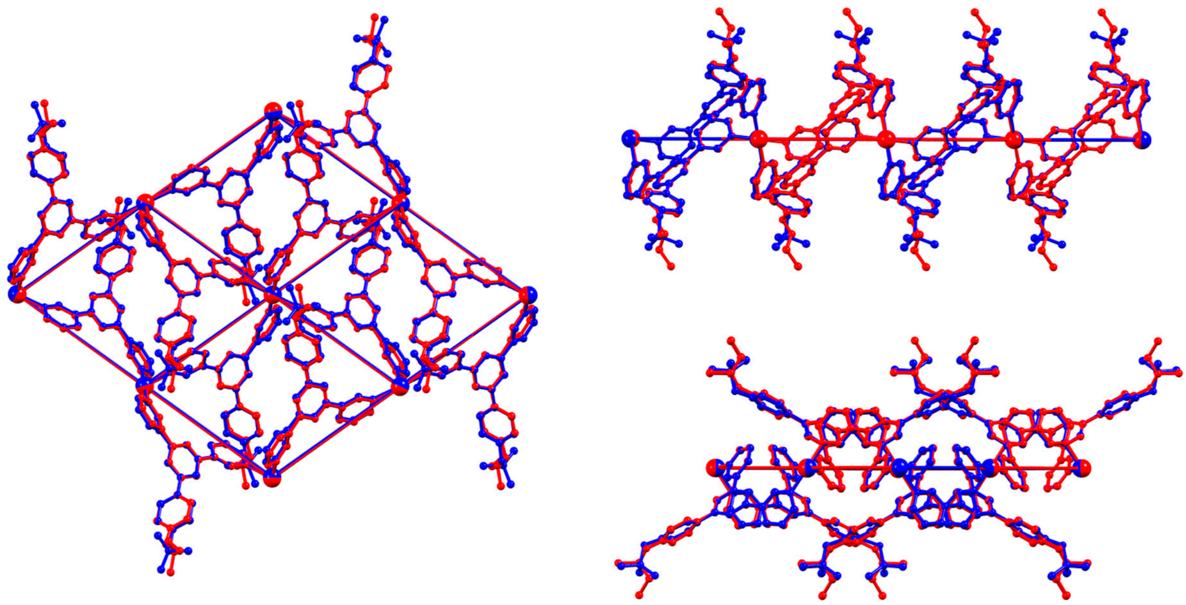

(a) (b)

Figure. S28. Overlays of parts of the (4,4) nets in [{Co(*rac*-**2**)$_2$(NCS)$_2$}·CHCl$_3$]$_n$ (red) and [{Co(**4**)$_2$(NCS)$_2$}·CHCl$_3$]$_n$ (blue).
